# Characterization of Nipah Virus from Outbreaks in Bangladesh, 2008–2010

**DOI:** 10.3201/eid1802.111492

**Published:** 2012-02

**Authors:** Michael K. Lo, Luis Lowe, Kimberly B. Hummel, Hossain M.S. Sazzad, Emily S. Gurley, M. Jahangir Hossain, Stephen P. Luby, David M. Miller, James A. Comer, Pierre E. Rollin, William J. Bellini, Paul A. Rota

**Affiliations:** Centers for Disease Control and Prevention, Atlanta, Georgia, USA (M.K. Lo, L. Lowe, K.B. Hummel, D.M. Miller, J.A. Comer, P.E. Rollin, W.J. Bellini, P.A. Rota);; International Centre for Diarrheal Disease Research, Bangladesh, Dhaka, Bangladesh (H.M.S. Sazzad, E.S. Gurley, M.J. Hossain, S.P. Luby)

**Keywords:** Nipah, Nipah virus, outbreak, encephalitis, phylogeny, viruses, Bangladesh

## Abstract

Nipah virus (NiV) is a highly pathogenic paramyxovirus that causes fatal encephalitis in humans. The initial outbreak of NiV infection occurred in Malaysia and Singapore in 1998–1999; relatively small, sporadic outbreaks among humans have occurred in Bangladesh since 2001. We characterized the complete genomic sequences of identical NiV isolates from 2 patients in 2008 and partial genomic sequences of throat swab samples from 3 patients in 2010, all from Bangladesh. All sequences from patients in Bangladesh comprised a distinct genetic group. However, the detection of 3 genetically distinct sequences from patients in the districts of Faridpur and Gopalganj indicated multiple co-circulating lineages in a localized region over a short time (January–March 2010). Sequence comparisons between the open reading frames of all available NiV genes led us to propose a standardized protocol for genotyping NiV; this protcol provides a simple and accurate way to classify current and future NiV sequences.

## Abstract 2

New genotyping scheme facilitates classification of virus sequences.

Nipah virus (NiV) is a deadly paramyxovirus that was first described during 1998–1999 in Malaysia and Singapore, when a large epidemic of fatal encephalitis occurred in humans (283 cases, 109 deaths) (*1*). In this initial outbreak, most human cases were epidemiologically linked with activities involving close contact with sick pigs; the outbreak ended after >1 million pigs were culled and movement of pigs was stopped (*2*). Although NiV infection has not been detected in Malaysia or Singapore since 1999, NiV has caused recurring (almost annual) outbreaks of fatal encephalitis in Bangladesh and sporadic outbreaks in India since 2001 (*3**–**6*). The outbreaks in Bangladesh have demonstrated human-to-human and foodborne transmission of NiV (*7**–**9*). Although the outbreaks in Bangladesh have been smaller, the case-fatality rates have been consistently higher (≈75%) than those from the initial outbreak in Malaysia and Singapore (≈40%) (*8**,**10*). The clinical case definition used in Bangladesh differs from that used during the Malaysia outbreak and focuses on fatal or severe neurologic signs and symptoms. Sequence analysis of virus isolates and clinical samples obtained from persons affected by the outbreaks in Bangladesh and India indicated greater nucleotide heterogeneity than those from Malaysia (*3**,**4**,**11*).

Within 2 weeks in Bangladesh during February 2008, 2 clusters of human NiV infection resulted in 10 cases with 9 deaths (90% case-fatality rate). The locations of the clusters (Rajbari and Manikgonj districts) were ≈44 km apart, separated by the intersection of the Padma and Jamuna Rivers. The outbreak was linked to ingestion of raw date palm sap (*12*). From December 2009 through March 2010, an outbreak of NiV infection in Fardipur and Gopalganj districts was responsible for 17 cases and 15 deaths (88% case-fatality rate) (*6*).

In this study, we confirmed the suspected clinical cases of NiV infection from both outbreaks by using IgM and IgG ELISAs, real-time and conventional reverse transcription PCR (RT-PCR), and virus isolation. We characterized the complete genomic sequences of 2 identical NiV isolates from 2008 and 3 partial genomic sequences of isolates from 2010. Our results indicate the presence of multiple co-circulating lineages of NiV in a localized region over a short time in 2010. Phylogenetic and sequence analysis of all currently available full-length NiV gene open reading frames (ORFs) led us to propose a standardized protocol for genotyping NiV.

## Methods

### Sample Collection and Case Definition

We collected blood, cerebrospinal fluid (CSF), urine, and throat swab samples from patients with suspected cases. The serum and CSF samples were separated into aliquots locally, and all specimens were transported to the International Centre for Diarrheal Disease Research, Bangladesh (ICDDR,B) in cold packs or in liquid nitrogen for subsequent storage at −70°C. Serum and CSF samples were initially tested for IgM against NiV at ICDDR,B and then sent to the Centers for Disease Control and Prevention (CDC) (Atlanta, GA, USA) for confirmatory testing. Samples were confirmed as NiV positive if IgM against NiV was found in serum or CSF; if NiV RNA was amplified; or if NiV was isolated from CSF, urine, or throat swab samples (*6**,**12*).

### Serologic Testing

Serum samples were tested at ICDDR,B for IgM against NiV by ELISA as described (*1**,**3**,**13*). At CDC, samples were irradiated with gamma rays before confirmatory testing for IgM and IgG against NiV as described (*3*).

### Detection of NiV by Real-Time, Conventional RT-PCR, and Virus Isolation

Virus isolation was attempted on Vero E6 cells as described (*1*). Human urine, CSF, and oropharyngeal swab samples were inactivated in guanidine isothiocynate (GITC) at a dilution of 1 part sample to 5 parts GITC. RNA was extracted by the acid GITC–phenol–chloroform method (*14*). Real-time RT-PCR (rRT-PCR) was performed by using the following primers that amplified a 112-nt fragment spanning from positions 538 to 650 in the NiV N gene: forward primer NVBNF2B 5′-CTGGTCTCTGCAGTTATCACCATCGA-3′, reverse primer NVBN593R 5′-ACGTACTTAGCCCATCTTCTAGTTTCA-3′, and probe NVBN542P 5′-CAGCTCCCGACACTGCCGAGGAT-3′, with the FAM dye incorporated at the 5′ terminus and a BHQ1 quencher molecule at the 3′ terminus. The rRT-PCR cycling conditions were as follows: 48°C for 30 min, 95°C for 10 min, and 45 cycles of 95°C for 15 s followed by 1 min at 60°C. Synthetic NiV N gene RNA was produced by in vitro transcription that used pTM1-N plasmid (*15*) with Megascript kit (Ambion, Austin, TX, USA) according to manufacturer’s instructions. An Applied Biosystems 7900HT machine was used for rRT-PCRs, and the PCR Core Kit along with MultiScribe Reverse Transcriptase were used for the rRT-PCR master mix (all from Applied Biosystems, Foster City, CA, USA). Conventional RT-PCR was performed with the SuperScript One-Step RT-PCR kit with Platinum Taq (Invitrogen, Carlsbad, CA, USA) as described (*11*). Two-step RT-PCR was performed for selected samples by using SuperScript III First-Strand Synthesis System (Invitrogen) to generate cDNA and Platinum Taq DNA Polymerase High Fidelity (Invitrogen) for the PCR. Briefly, 8 μL of extracted RNA was used in a 20-μL RT reaction with a primer complementary to the 3′ leader NVB3END (5′-ACCAAACAAGGGAAAATATGGATACGTT-3′) and the 5′ trailer NIP5END (5′-ACCGAACAAGGGTAAAGAAGAATCG-3′) sequences of the NiV genome from the 2004 Bangladesh outbreak (GenBank accession no. AY988601). Subsequently, 2 μL of the cDNA reaction was used in 50-μL PCRs to amplify the N, P, M, F, G, and L ORFs with corresponding primer sets that anneal to the noncoding regions for the respective genes: N ORF NVBN5NCF1 (5′-GGTCTTGGTATTGGATCCTC-3′) and NVBN3NCR1 (5′-GTTTAATCTAAGTTAAGATTG-3′); P ORF NVBPPCRFW (5′-AGCAGTTATCAGCTGGGAGTTCAACTTAC-3′) and NVBPPCRREV (5′-ATGCGTGAATGAACTACAATACGAATCGAC-3′); M ORF NVBMPCRFW (5′-TCCAATAACTGGTCAATTGAGGACAGAAATCCTG-3′) and NVBMPCRREV (5′-CATAATAGTTGTCTAATTATTAACCGAATATTCAC-3′); F ORF NVBPCRFFW (5′-CAAGCATTATTACTATCTGATCAACAAAAGGATTGG-3′) and NVBPCRFREV (5′-GAATATCAACTGTTCATTCATGGTTGAGTAC-3′); G ORF NVBPCRGFW (5′-CAGGTCCATAACTCATTGGATATTAAACTGTGTCC-3′) and NVBPCRGREV (5′-CAAGATTTAGCTCTACTATATCAAATGGAGTTTCAGTCAAG-3′); and L ORF (amplified in 2 fragments) NVBPCRL1FW (5′-CAGGTCCTTGATTGTGCTAATTTTCTTGAG-3′) and FRAG4REV (5′-GATCTTATCAGGCCTTTAGTTGTATCTAATAGACC-3′), FRAG5FW (5′-TGAGGACCTTGAACTAGCTAGCTTCCT-3′) and NVBLREV (5′-AATTGTCGGTCGGTTCTGGACTTGGAAGATCAAATCAGATAATGGATATG-3′). PCR products were analyzed by agarose gel electrophoresis with GelRed staining (Biotium, Hayward, CA, USA), gel purified, and sequenced as described (*3**,**11*). Rapid amplification of cDNA ends was performed by using the 5′ RACE Kit 2.0 (Invitrogen). Phylogenetic and molecular analyses of the sequences were performed by using MEGA5 (*16*).

## Results

**Figure 1:**
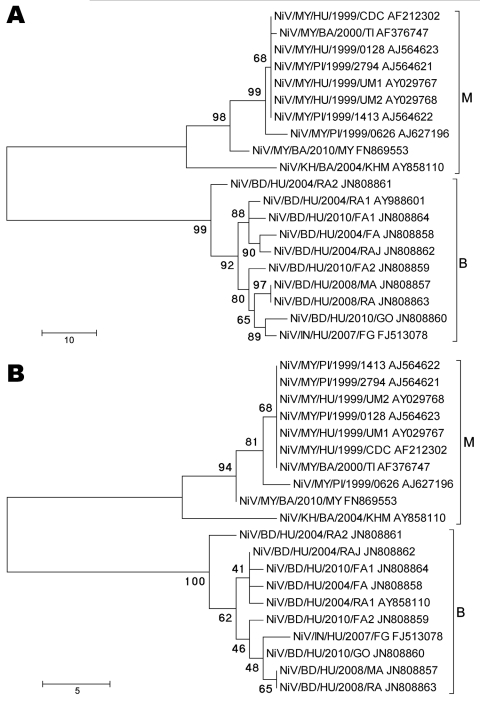
Phylogenetic analyses of sequences from the complete Nipah virus N ORF (A) and the 729-nt proposed N ORF genotyping window (B). Tree created with maximum parsimony, close-neighbor-interchange algorithm, 1,000 bootstrap replicates (*16*). Branch lengths are in units of number of changes over the whole sequence. Available GenBank accession numbers are shown for corresponding sequences. Proposed genotype groupings are indicated by brackets (M, B). ORF, open reading frame; MY, Malaysia; KH, Cambodia; BD, Bangladesh; IN, India; HU, human; PI, pig; BA, bat. Scale bars indicate number of sequence changes corresponding to illustrated branch length.

**Figure 2:**
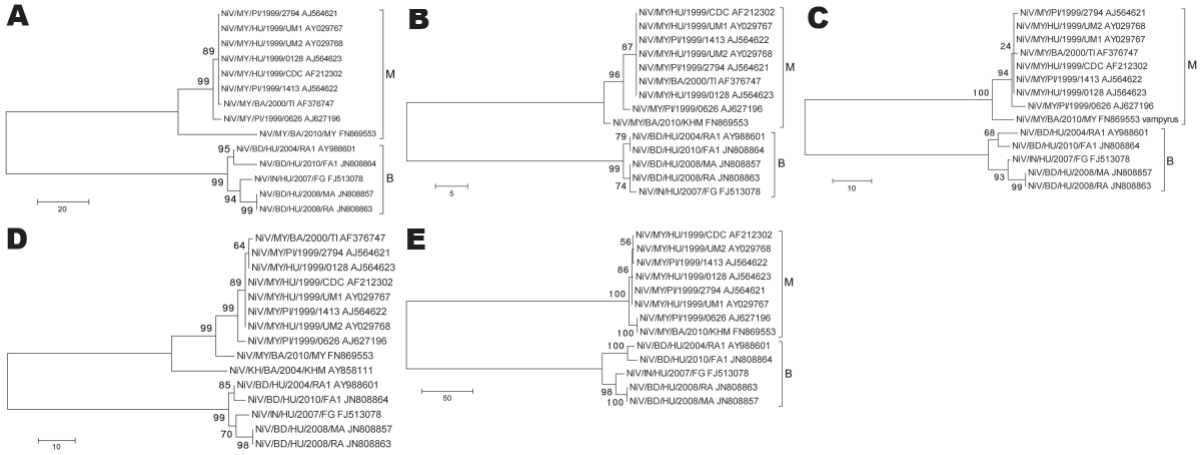
Phylogenetic analyses of sequences from the complete Nipah virus P ORF (A), M ORF (B), F ORF (C), G ORF (D), and L ORF (E). Tree created with maximum parsimony, close-neighbor-interchange algorithm, 1,000 bootstrap replicates (*16*). Branch lengths are in units of number of changes over the whole sequence. Available GenBank accession numbers are shown for corresponding sequences. Proposed genotype groupings are indicated by brackets (M, B). ORF, open reading frame; MY, Malaysia; KH, Cambodia; BD, Bangladesh; IN, India; HU, human; PI, pig; BA, bat. Scale bars indicate number of sequence changes corresponding to illustrated branch length.

Of the 10 cases from the 2008 outbreak in Bangladesh, 5 were confirmed positive for NiV infection by at least 1 laboratory test at CDC. Of those 5 positive cases, 4 were positive for IgM; 2 for IgG; 3 by rRT-PCR; and 2 by conventional RT-PCR; 2 throat swab samples yielded live NiV, 1 from Manikgonj (NiV/BD/HU/2008/MA [BD, Bangladesh; HU, human]; accession no. JN808857) and 1 from Rajbari (NiV/BD/HU/2008/RA; accession no. JN808863) (Table 1). Despite the isolates having come from patients from 2 districts, sequence analysis of the entire genome of the 2 isolates indicated that they were identical. Phylogenetic analysis of ORFs from each NiV gene indicated that this strain was similar to, but distinct from, the 2007 isolate from India (NiV/IN/HU/2007/FG [IN, India]; accession no. FJ513078) (Figure 1, panel A; Figure 2, panels A–E). To rule out the possibility of laboratory contamination, we performed 2-step conventional RT-PCR by using RNA from duplicate samples of the original throat swab samples from which the 2 viruses were isolated. We amplified the entire N gene ORF from each sample and confirmed that the sequences were identical. Although there were 4 isolated cases of NiV infection in Bangladesh in 2009 as confirmed by IgM or IgG ELISA, or both, we were not able to obtain NiV sequences from those case-patients (Table 1).

**Table 1:** Results from patients with confirmed Nipah virus infection, Bangladesh, 2008–2010*

Patient no.	Year isolated	Case type	Serologic result		RT-PCR result		Virus isolation
			IgM	IgG	Conventional	Real-time	
1	2008	Cluster	+	+	–	–	–
2	2008	Cluster	+	–	–	+	–
3	2008	Cluster	–	–	+	+	+
4	2008	Cluster	+	–	+	+	+
5	2008	Cluster	+	+	–	–	–
6	2009	Isolated	+	+	NA	NA	NA
7	2009	Isolated	+	–	NA	NA	NA
8	2009	Isolated	+	–	NA	NA	NA
9	2009	Isolated	+	–	NA	NA	NA
10	2010	Cluster	+	–	NA	NA	NA
11	2010	Cluster	+	–	+	+	–
12	2010	Cluster	+	–	–	+	–
13	2010	Cluster	+	–	NA	NA	NA
14	2010	Cluster	+	+	NA	NA	NA
15	2010	Isolated	+	–	–	+	–
16	2010	Isolated	+	+	+	+	–
17	2010	Isolated	+	–	+	+	–
18	2010	Isolated	+	–	NA	NA	NA
19	2010	Isolated	+	–	NA	NA	NA
20	2010	Isolated	+	–	NA	NA	NA
21	2010	Isolated	+	–	NA	NA	NA

*RT-PCR, reverse transcription PCR; +, positive; **–,** negative; NA, sample not available.

Of the 17 cases from the 2010 outbreak in Bangladesh, 12 were confirmed positive. All 12 were positive for IgM, 2 for IgG, 5 by rRT-PCR, and 3 by conventional 2-step RT-PCR (Table 1). Although we detected NiV RNA by rRT-PCR from urine, CSF, and throat swab samples, we were unable to isolate virus from any of those sources. Of the 3 samples from which we were able to amplify NiV sequences, 1 was from a 10-year-old girl from the initial cluster (NiV/BD/HU/2010/FA2; accession no. JN808859) and the other 2 were from patients with isolated cases. The patients with isolated cases were a medical intern (NiV/BD/HU/2010/FA1; accession no. JN808864) who was working in the pediatric department at Faridpur Medical College Hospital and a 7-year-old girl (NiV/BD/HU/2010/GO; accession no. JN808860) who was examined by the same medical intern; both died. The illness in the intern developed only 6 days after the 7-year-old girl died; this incubation period was atypically short for NiV infection, indicating the possibility of separate infections (*6*). Sequence analysis of the N ORFs amplified from throat swab samples confirmed that the intern and the girl were infected with distinct lineages of NiV (Figure 1, panel A). Our attempts to recover NiV sequences from prior contacts of the medical intern who were IgM positive for NiV infection were unsuccessful. Phylogenetic analysis indicated that the sequence from the 7-year-old girl was similar to, but distinct from, the 2007 isolate from India, whereas the sequence from the intern was closer to that of the 2004 isolate from Bangladesh (NiV/BD/HU/2004/RA1; accession no. AY988601). The N sequence obtained from the 10-year-old girl from the initial cluster was shown to be slightly more similar to the 2007 isolate from India (Figure 1, panel A). We were only able to amplify the complete N ORF from the throat swab samples from the 7-year-old and 10-year-old girls because our rRT-PCR indicated the presence of ≈10^3^ to 10^4^ copies of NiV N RNA (cycle threshold ≈26–30). The rRT-PCR conducted on the throat swab sample from the medical intern indicated the presence of ≈10^6^ copies of NiV N RNA (cycle threshold ≈20), which corroborated our ability to amplify nearly the entire genome except for the 3′ leader and 5′ trailer (data not shown) from this sample.

Since the initial molecular characterization of NiV from Bangladesh in 2004 (*11*), there has been a shortage of full-length NiV ORF sequences from Bangladesh. However, the sequence data obtained from the 2008 and 2010 Bangladesh outbreaks in this study, along with the recent characterization of the 2007 isolate from India (*4*), support the previous observation of relative heterogeneity among NiV nucleotide sequences from humans affected by outbreaks in Bangladesh compared with sequences from Malaysia (*11*). Phylogenetic analysis indicated that these new sequences from Bangladesh and India group substantially closer to the sequences from Bangladesh in 2004, which led us to propose a system to describe the distinct lineages of NiV (Figure 1; Figure 2, panels A–E). We propose to designate the current sequences obtained from Malaysia (MY) and Cambodia (KH) as genotype M and the sequences obtained from Bangladesh and India as genotype B. By using a 729-nt window in the N terminal region of the N gene ORF (N ORF nt 123–852, NiV genome positions 236–964), we were able to determine 25 distinct nucleotides that universally differentiated the genotypes (Figure 1, panel B). The topology of the phylogenetic tree and the positions of the branches generated from this smaller nucleotide window were similar to those of the tree generated with the full-length N ORF sequences and have reasonably high bootstrap values at the root branch junctions, albeit with lower bootstrap values at the distal branch junctions (Figure 1, panels A, B). In support of this scheme, we observed similar topologies and branching patterns in phylogenetic trees generated for the complete P, M, F, G, and L ORFs, all with strong bootstrap values (Figure 2, panels A–E).

Pairwise sequence comparisons conducted across each individual NiV gene ORF indicated a nucleotide variation range of 6.32%–9.15% between genotype M and B viruses and an amino acid variation range of 1.42%–9.87% (Table 2; Technical Appendix). The ranges of nucleotide and amino acid variation of sequences within genotype M were 0.19%–2.21% and 0.18–3.67%, respectively, and within genotype B were 0.28%–1.06% and 0.28% –0.56%, respectively. The apparently higher levels of variation found among ORFs within genotype M is mostly caused by the comparatively divergent sequences obtained from *Pteropus vampyrus* (NiV/MY/BA/2010/MY; accession no. FN869553) and *P. lylei* (NiV/KH/BA/2004/KHM; accession nos. AY858110, AY858111) bats. Not only is the proposed genotyping scheme supported by consistent phylogenetic tree topologies, but pairwise nucleotide comparisons of the 729-nt region yield similar percentages of variability as seen in the full-length N ORF comparisons. This finding indicates that this sequence window is a relatively accurate indicator of overall nucleotide variability within and across genotypes M and B (Technical Appendix Figure 1, panels A, C).

**Table 2:** Percentage nucleotide and amino acid variability among available complete Nipah virus open reading frame sequences

Gene	Open reading frame length, nt/aa	% nt variation				% aa variation		
		Overall	Genotype M	Genotype B		Overall	Genotype M	Genotype B
N	1,599/532	0.0–6.32	0.0–2.19	0.0–1.06		0.0–2.26	0.0–1.69	0.0–0.56
P	2,130/709	0.0–9.15	0.0–2.21	0.0–0.99		0.0–9.87	0.0–3.67	0.0–0.99
M	1,059/352	0.0–6.70	0.0–0.57	0.0–0.28		0.0–1.42	0.0–0.85	0.0–0.28
F	1,641/546	0.0–6.76	0.0–0.85	0.0–0.98		0.0–1.65	0.0–0.75	0.0–0.55
G	1,809/602	0.0–7.35	0.0–1.93	0.0–0.55		0.0–4.65	0.0–1.83	0.0–0.33
L	6,735/2244	0.0–6.68	0.01–0.19	0.0–0.82		0.0–1.92	0.0–0.18	0.0–0.45

A comprehensive amino acid alignment of currently available complete N protein ORFs indicated that the 4 residues that distinguish between genotype M and B viruses are almost all located in the COOH-terminus (Table 3). Of these residues, only 1 (position 387) is located within the putative minimum contiguous sequence required for capsid assembly (*17*), and none were located in the 29 COOH-terminal and 10 NH-terminal residues required for interaction with the P protein (*18**,**19*). Of note, there are 4 residues (V429, E432, D457, and T521) in the COOH-terminal region common to all genotype B sequences that are shared with 2 of the comparatively divergent genotype M sequences. In light of the overall nucleotide and amino acid sequence comparisons, however, the divergent genotype M sequences from the bats still differ substantially from genotype B sequences (Figure 1, panel A; Figure 2, panels A–E; Technical Appendix Figure 1, panels A, B).

**Table 3:** Amino acid differences among available complete Nipah virus N gene open reading frame sequences*

Sequence and accession no.	Amino acid position																					
	G	30	139	188	211	318	345	380	381	387	414	429	432	436	457	502	505	506	508	511	518	521
NiV/MY/HU/1999/CDC, AF212302	M	T	S	E	Q	I	M	N	R	D	K	I	G	I	N	I	R	T	G	E	L	A
NiV/MY/PI/1999/1413, AJ564622	M	.	.	.	.	.	.	.	.	.	.	.	.	.	.	.	.	.	.	.	.	.
NiV/MY/PI/1999/2794, AJ564621	M	.	.	.	.	.	.	.	.	.	.	.	.	.	.	.	.	.	.	.	.	.
NiV/MY/PI/1999/0626, AJ627196	M	.	R	.	.	.	I	.	.	.	.	.	.	.	.	.	.	.	.	.	.	.
NiV/MY/HU/1999/0128, AJ564623	M	.	.	.	.	.	.	.	.	.	.	.	.	.	.	.	.	.	.	.	.	.
NiV/MY/HU/1999/UM1, AY029767	M	.	.	.	.	.	.	.	.	.	.	.	.	.	.	.	.	.	.	.	.	.
NiV/MY/HU/1999/UM2, AY029768	M	.	.	.	.	.	.	.	.	.	.	.	.	.	.	.	.	.	.	.	.	.
NiV/MY/BA/2000/TI, AF376747	M	I	.	.	.	.	.	.	.	.	.	.	.	.	.	.	.	.	.	.	.	.
NiV/MY/BA/2010/MY, FN869553	M	.	.	.	.	.	.	.	.	.	.	V	E	.	D	.	.	.	.	.	.	.
NiV/KH/BA/2004/KHM, AY858110	M	.	.	.	.	.	.	.	.	.	.	V	E	.	D	T	.	.	.	G	P	T
NiV/BD/HU/2004/1, AY988601	B	.	.	D	.	.	.	.	.	N	.	V	E	.	D	.	K	D	R	.	.	T
NiV/BD/HU/2004/FA, JN808858	B	.	.	.	.	.	.	.	.	N	N	V	E	M	D	.	K	D	R	.	.	T
NiV/BD/HU/2004/RA2, JN808861	B	.	.	.	.	.	.	.	.	N	.	V	E	.	D	.	K	D	R	.	.	T
NiV/BD/HU/2004/RAJ, JN808862	B	.	.	.	.	.	.	I	.	N	.	V	E	M	D	.	K	D	R	.	.	T
NiV/BD/HU/2008/MA, JN808857	B	.	.	.	.	.	.	.	.	N	.	V	E	.	D	.	K	D	R	.	.	T
NiV/BD/HU/2008/RA, JN808863	B	.	.	.	.	.	.	.	.	N	.	V	E	.	D	.	K	D	R	.	.	T
NiV/BD/HU/2010/FA1, JN808864	B	.	.	.	.	.	.	.	K	N	.	V	E	.	D	.	K	D	R	.	.	T
NiV/BD/HU/2010/GO, JN808860	B	.	.	.	.	V	.	.	.	N	.	V	E	.	D	.	K	D	R	.	.	T
NiV/BD/HU/2010/FA2, JN808859	B	.	.	.	.	.	.	.	.	N	.	V	E	.	D	.	K	D	R	.	.	T
NiV/IN/HU/2007/FG, FJ513078	B	.	.	.	R	.	.	.	.	N	.	V	E	.	D	.	K	D	R	.	.	T

*Dots indicate sequence identity with AF212302. NiV, Nipah virus; MY, Malaysia; HU, human; G, genotype classification; genotype M, sequences from Malaysia and Cambodia; genotype B, sequences from Bangladesh and India; T, threonine, S, serine; E, glutamate; Q, glutamine; I, isoleucine; M, methionine; N, asparagine; R, arginine; D, aspartate; K, lysine; G, glycine; V; valine; P, proline; PI, pig; BA, bat; KH, Cambodia; BD, Bangladesh; IN, India.

Amino acid alignments of the P protein indicated numerous differences between genotype M and B sequences in the first 400 residues, which comprise the shared N terminal region between the P, V, and W proteins. Of the differences in this region, there were neither changes that would be predicted to alter the STAT-1 binding ability of the P, V, and W proteins nor changes that could adversely affect RNA replication (*20**–**22*). There were only 4 changes in the COOH-terminal region of P, which is required for direct N–P interactions, 2 of which were nonconservative changes (N590→S, E635→G) (*18*). The P sequence derived from *P. vampyrus* bats had an intriguing sequence of amino acids from residues 408–440, in which there was substantial sequence divergence from genotypes M and B at the nucleotide and amino acid levels (*23*). These particular nucleotide changes in the P sequence also introduced several amino acid changes in the unique COOH-terminal regions of the V (11 changes) and W (9 changes) ORFs, which distinguish them from any genotype M and B sequences.

We observed the M protein to be highly conserved across genotypes M and B, and we found just 2 aa residues exclusive to genotype B that are not located in any region of the protein with a known function, such as budding (*24**,**25*), nuclear localization, or ubiquitination (*26*). In the F protein, the predicted cleavage site, F1 amino-terminal domain, transmembrane domain, and predicted N-linked glycosylation sites are all conserved across both genotypes. Although the percentage of amino acid variation in the G protein is higher than that in all other NiV proteins (except the P protein), it is not surprising that the residues implicated in Ephrin B2 and B3 binding are conserved across the genotypes (*27**,**28*). The amino acid differences between genotypes M and B sequences are predominantly found at residues that are distant from the receptor binding site. Two differences were found in the intracellular domain, 3 differences in the stalk region (positions 72–182), 9 differences in a span of ≈100 aa (positions 236–344) along the side of the globular head domain, 4 differences closer to the top of the globular head domain (positions 385–424), and only 2 differences (positions 498 and 502) that were close to the tryptophan residue at position 504, which is part of the receptor-binding pocket. As in other NiV proteins, several amino acids were shared between genotype B sequences and 2 genotype M sequences derived from the bat isolates. The significance of these changes has yet to be explored.

The level of amino acid conservation throughout the L proteins was high; the purported GDNE catalytic site and the K-X_21_-GEGSG ATP binding site were conserved across genotypes M and B. Most distinct differences between genotypes M and B sequences (26 of 32) were located outside the 6 linear domains typically found in nonsegmented negative strand virus polymerases (*29**,**30*). The cis-acting control sequences in NiV are usually well conserved. The tri-nucleotide intergenic sequences amplified from the throat swab sample from the medical intern in 2010 had GAA for all 6 intergenic regions, which was identical to the 2007 isolate from India. For the 2008 isolates, the intergenic sequence between the N and P genes was AAA, and the rest of the intergenic sequences were GAA. The biological implications of finding adenosine in the first position of NiV intergenic sequences is unknown. The 3′ leader and 5′ trailer sequences of the 2008 NiV isolates were identical to those found in the 2004 Bangladesh and 2007 India isolates.

## Discussion

From the initial outbreak of NiV in Malaysia until now, there has not been a standard method by which to classify NiVs. With the accumulation of sequences from subsequent human outbreaks in Bangladesh and India, along with an increasing number of bat-derived sequences, we propose a standardized genotyping method for NiV. The goal behind a genotyping scheme is to classify viruses by using a smaller sequence window that has levels of sequence variability that correspond to variability between complete genomes and that would give the same phylogenetic tree topology with high bootstrap values. Genotyping schemes for other paramyxoviruses, such as measles virus and mumps virus, have been delineated (*31**,**32*).

Before this study, there has been a growing body of partial-sequence data obtained from a 357-nt region coding for the COOH-terminus of N (NiV genome positions 1197–1553) (*23**,**33*). Although obtaining sequence information from this window has the advantage of tracking more variability at the nucleotide and the amino acid levels, it could potentially overestimate the level of variability between sequences within and across genotypes. Pairwise nucleotide sequence comparisons performed by using the 357-nt window estimate the overall sequence variation at ≈8%, whereas the sequence variation of the complete N ORF is ≈6% (Table 2; Technical Appendix Figure 1, panels A, D). In particular, the 357-nt window overestimates the variability within genotype M of the *P. vampyrus* bat sequence at ≈2%, whereas variability of the sequence within genotype M is <1% when the complete N ORF is taken into account. The 729-nt window in the N terminal region proposed in this study would serve as a more conservative scheme for genotyping because it has little amino acid variation but has nucleotide variability of ≈5.5% according to pairwise comparisons between the 2 proposed genotypes, which only slightly underestimates the variability among sequences for the complete N ORF (Technical Appendix Figure 1, panels A, C). As with other genotyping schemes that facilitate the classification of viruses, our proposed scheme is amenable to corrective measures as warranted by evidence from sequences obtained from future outbreaks and bat surveillance studies.

In summary, we conducted a comprehensive molecular phylogenetic analysis of currently available complete NiV gene ORFs at the nucleotide and amino acid levels, including newly obtained sequence data from NiV outbreaks in Bangladesh in 2008 and 2010. Analyses of the combined sequence data obtained from Bangladesh and India in the past decade led us to propose a genotyping scheme based on a 729-nt window of the NiV N ORF. This genotyping scheme provides a simple and accurate way to classify current and future NiV sequences.

## Supplementary Material

Technical Appendix Pairwise sequence comparisons conducted across individual Nipah virus gene open reading frames.
