# Supplementary material for: Characterization of Nipah Virus from Outbreaks in Bangladesh, 2008–2010
Source: Emerg Infect Dis. 2012 Feb;18(2):248–55. doi: 10.3201/eid1802.111492 (PMC3310473; doi:10.3201/eid1802.111492)
Supplement: Technical Appendix — Pairwise sequence comparisons conducted across individual Nipah virus gene open reading frames. [file 11-1492-Techapp_15p.pdf]

Article DOI: http://dx.doi.org/10.3201/eid1802.111492

Characterization of Nipah Virus from Outbreaks in Bangladesh, 2008–2010

Technical Appendix

The following pages show estimates of evolutionary divergence between Nipah virus (NiV) open reading frame (ORF) nucleotide and amino acid sequences. All positions containing gaps and missing data were eliminated. All evolutionary analyses were conducted by using MEGA5 (*1*). MY, Malaysia; KH, Cambodia; BD, Bangladesh; IN, India; HU, human; PI, pig; BA, bat.

Reference

<jrn>1. Tamura K, Peterson D, Peterson N, Stecher G, Nei M, Kumar S. MEGA5: Molecular Evolutionary Genetics Analysis using maximum likelihood, evolutionary distance, and maximum parsimony methods. Mol Biol Evol. 2011. Submitted. Medline doi:10.1093/molbev/msr121</jrn>

Nipah sequence ID	Accession #	Genotype																			
NiV/MY/HU/1999/CDC	AF212302	M																			
NiV/MY/PI/1999/1413	AJ564622	M	0.0																		
NiV/MY/PI/1999/2794	AJ564621	M	0.0	0.0																	
NiV/MY/HU/1999/0128	AJ564623	M	0.0	0.0	0.0																
NiV/MY/HU/1999/UM1	AY029767	M	0.0	0.0	0.0	0.0															
NiV/MY/HU/1999/UM2	AY029768	M	0.0	0.0	0.0	0.0	0.0														
NiV/MY/BA/2000/TI	AF376747	M	1.0	1.0	1.0	1.0	1.0	1.0													
NiV/MY/PI/1999/0626	AJ627196	M	5.0	5.0	5.0	5.0	5.0	5.0	6.0												
NiV/MY/BA/2010/MY	FN869553	M	11.0	11.0	11.0	11.0	11.0	11.0	12.0	14.0											
NiV/KH/BA/2004/KHM	AY858110	M	32.0	32.0	32.0	32.0	32.0	32.0	33.0	35.0	27.0										
NiV/BD/HU/2004/RA1	AY988601	B	91.0	91.0	91.0	91.0	91.0	91.0	92.0	94.0	90.0	95.0									
NiV/BD/HU/2004/FA	JN808858	B	95.0	95.0	95.0	95.0	95.0	95.0	96.0	98.0	94.0	99.0	7.0								
NiV/BD/HU/2004/RA2	JN808861	B	88.0	88.0	88.0	88.0	88.0	88.0	89.0	91.0	85.0	90.0	12.0	15.0							
NiV/BD/HU/2004/RAJ	JN808862	B	94.0	94.0	94.0	94.0	94.0	94.0	95.0	97.0	93.0	98.0	6.0	5.0	14.0						
NiV/BD/HU/2008/MA	JN808857	B	90.0	90.0	90.0	90.0	90.0	90.0	91.0	93.0	89.0	94.0	10.0	13.0	14.0	12.0					
NiV/BD/HU/2008/RA	JN808863	B	90.0	90.0	90.0	90.0	90.0	90.0	91.0	93.0	89.0	94.0	10.0	13.0	14.0	12.0	0.0				
NiV/BD/HU/2010/FA1	JN808864	B	91.0	91.0	91.0	91.0	91.0	91.0	92.0	94.0	90.0	95.0	5.0	8.0	13.0	7.0	11.0	11.0			
NiV/BD/HU/2010/GO	JN808860	B	97.0	97.0	97.0	97.0	97.0	97.0	98.0	98.0	96.0	101.0	13.0	16.0	17.0	15.0	9.0	9.0	14.0		
NiV/BD/HU/2010/FA2	JN808859	B	91.0	91.0	91.0	91.0	91.0	91.0	92.0	94.0	90.0	97.0	9.0	12.0	13.0	11.0	7.0	7.0	10.0	10.0	
NiV/IN/HU/2007/FG	FJ513078	B	95.0	95.0	95.0	95.0	95.0	95.0	96.0	98.0	94.0	99.0	9.0	12.0	15.0	11.0	7.0	7.0	10.0	6.0	8.0

Nipah sequence ID	Accession #	Genotype																			
NiV/MY/HU/1999/CDC	AF212302	M																			
NiV/MY/PI/1999/1413	AJ564622	M	0.00%																		
NiV/MY/PI/1999/2794	AJ564621	M	0.00%	0.00%																	
NiV/MY/HU/1999/0128	AJ564623	M	0.00%	0.00%	0.00%																
NiV/MY/HU/1999/UM1	AY029767	M	0.00%	0.00%	0.00%	0.00%															
NiV/MY/HU/1999/UM2	AY029768	M	0.00%	0.00%	0.00%	0.00%	0.00%														
NiV/MY/BA/2000/TI	AF376747	M	0.06%	0.06%	0.06%	0.06%	0.06%	0.06%													
NiV/MY/PI/1999/0626	AJ627196	M	0.31%	0.31%	0.31%	0.31%	0.31%	0.31%	0.38%												
NiV/MY/BA/2010/MY	FN869553	M	0.69%	0.69%	0.69%	0.69%	0.69%	0.69%	0.75%	0.88%											
NiV/KH/BA/2004/KHM	AY858110	M	2.00%	2.00%	2.00%	2.00%	2.00%	2.00%	2.06%	2.19%	1.69%										
NiV/BD/HU/2004/RA1	AY988601	B	5.69%	5.69%	5.69%	5.69%	5.69%	5.69%	5.75%	5.88%	5.63%	5.94%									
NiV/BD/HU/2004/FA	JN808858	B	5.94%	5.94%	5.94%	5.94%	5.94%	5.94%	6.00%	6.13%	5.88%	6.19%	0.44%								
NiV/BD/HU/2004/RA2	JN808861	B	5.50%	5.50%	5.50%	5.50%	5.50%	5.50%	5.57%	5.69%	5.32%	5.63%	0.75%	0.94%							
NiV/BD/HU/2004/RAJ	JN808862	B	5.88%	5.88%	5.88%	5.88%	5.88%	5.88%	5.94%	6.07%	5.82%	6.13%	0.38%	0.31%	0.88%						
NiV/BD/HU/2008/MA	JN808857	B	5.63%	5.63%	5.63%	5.63%	5.63%	5.63%	5.69%	5.82%	5.57%	5.88%	0.63%	0.81%	0.88%	0.75%					
NiV/BD/HU/2008/RA	JN808863	B	5.63%	5.63%	5.63%	5.63%	5.63%	5.63%	5.69%	5.82%	5.57%	5.88%	0.63%	0.81%	0.88%	0.75%	0.00%				
NiV/BD/HU/2010/FA1	JN808864	B	5.69%	5.69%	5.69%	5.69%	5.69%	5.69%	5.75%	5.88%	5.63%	5.94%	0.31%	0.50%	0.81%	0.44%	0.69%	0.69%			
NiV/BD/HU/2010/GO	JN808860	B	6.07%	6.07%	6.07%	6.07%	6.07%	6.07%	6.13%	6.13%	6.00%	6.32%	0.81%	1.00%	1.06%	0.94%	0.56%	0.56%	0.88%		
NiV/BD/HU/2010/FA2	JN808859	B	5.69%	5.69%	5.69%	5.69%	5.69%	5.69%	5.75%	5.88%	5.63%	6.07%	0.56%	0.75%	0.81%	0.69%	0.44%	0.44%	0.63%	0.63%	
NiV/IN/HU/2007/FG	FJ513078	B	5.94%	5.94%	5.94%	5.94%	5.94%	5.94%	6.00%	6.13%	5.88%	6.19%	0.56%	0.75%	0.94%	0.69%	0.44%	0.44%	0.63%	0.38%	0.50%

Technical Appendix Figure 1A. Estimates of evolutionary divergence between NiV N ORF nucleotide sequences. The number of base differences (top matrix) and percentage of difference (bottom matrix) per sequence from between sequences are shown. The analysis involved 20 nt sequences. Codon positions included were 1st+2nd+3rd+noncoding. There were a total of 1,599 positions in the final dataset.

Nipah sequence ID	Accession #	Genotype																			
NiV/MY/HU/1999/CDC	AF212302	M																			
NiV/MY/PI/1999/1413	AJ564622	M	0.0																		
NiV/MY/PI/1999/2794	AJ564621	M	0.0	0.0																	
NiV/MY/HU/1999/0128	AJ564623	M	2.0	2.0	2.0																
NiV/MY/HU/1999/UM1	AY029767	M	0.0	0.0	0.0	2.0															
NiV/MY/HU/1999/UM2	AY029768	M	0.0	0.0	0.0	2.0	0.0														
NiV/MY/BA/2000/TI	AF376747	M	0.0	0.0	0.0	2.0	0.0	0.0													
NiV/MY/PI/1999/0626	AJ627196	M	1.0	1.0	1.0	3.0	1.0	1.0	1.0												
NiV/MY/BA/2010/MY	FN869553	M	3.0	3.0	3.0	5.0	3.0	3.0	3.0	4.0											
NiV/KH/BA/2004/KHM	AY858110	M	7.0	7.0	7.0	9.0	7.0	7.0	7.0	8.0	4.0										
NiV/BD/HU/2004/RA1	AY988601	B	9.0	9.0	9.0	11.0	9.0	9.0	9.0	10.0	6.0	8.0									
NiV/BD/HU/2004/FA	JN808858	B	10.0	10.0	10.0	12.0	10.0	10.0	10.0	11.0	7.0	9.0	3.0								
NiV/BD/HU/2004/RA2	JN808861	B	8.0	8.0	8.0	10.0	8.0	8.0	8.0	9.0	5.0	7.0	1.0	2.0							
NiV/BD/HU/2004/RAJ	JN808862	B	10.0	10.0	10.0	12.0	10.0	10.0	10.0	11.0	7.0	9.0	3.0	2.0	2.0						
NiV/BD/HU/2008/MA	JN808857	B	8.0	8.0	8.0	10.0	8.0	8.0	8.0	9.0	5.0	7.0	1.0	2.0	0.0	2.0					
NiV/BD/HU/2008/RA	JN808863	B	8.0	8.0	8.0	10.0	8.0	8.0	8.0	9.0	5.0	7.0	1.0	2.0	0.0	2.0	0.0				
NiV/BD/HU/2010/FA1	JN808864	B	9.0	9.0	9.0	11.0	9.0	9.0	9.0	10.0	6.0	8.0	2.0	3.0	1.0	3.0	1.0	1.0			
NiV/BD/HU/2010/GO	JN808860	B	9.0	9.0	9.0	11.0	9.0	9.0	9.0	10.0	6.0	8.0	2.0	3.0	1.0	3.0	1.0	1.0	2.0		
NiV/BD/HU/2010/FA2	JN808859	B	8.0	8.0	8.0	10.0	8.0	8.0	8.0	9.0	5.0	7.0	1.0	2.0	0.0	2.0	0.0	0.0	1.0	1.0	
NiV/IN/HU/2007/FG	FJ513078	B	9.0	9.0	9.0	11.0	9.0	9.0	9.0	10.0	6.0	8.0	2.0	3.0	1.0	3.0	1.0	1.0	2.0	2.0	1.0

Nipah sequence ID	Accession #	Genotype																			
NiV/MY/HU/1999/CDC	AF212302	M																			
NiV/MY/PI/1999/1413	AJ564622	M	0.00%																		
NiV/MY/PI/1999/2794	AJ564621	M	0.00%	0.00%																	
NiV/MY/HU/1999/0128	AJ564623	M	0.38%	0.38%	0.38%																
NiV/MY/HU/1999/UM1	AY029767	M	0.00%	0.00%	0.00%	0.38%															
NiV/MY/HU/1999/UM2	AY029768	M	0.00%	0.00%	0.00%	0.38%	0.00%														
NiV/MY/BA/2000/TI	AF376747	M	0.00%	0.00%	0.00%	0.38%	0.00%	0.00%													
NiV/MY/PI/1999/0626	AJ627196	M	0.19%	0.19%	0.19%	0.56%	0.19%	0.19%	0.19%												
NiV/MY/BA/2010/MY	FN869553	M	0.56%	0.56%	0.56%	0.94%	0.56%	0.56%	0.56%	0.75%											
NiV/KH/BA/2004/KHM	AY858110	M	1.32%	1.32%	1.32%	1.69%	1.32%	1.32%	1.32%	1.50%	0.75%										
NiV/BD/HU/2004/RA1	AY988601	B	1.69%	1.69%	1.69%	2.07%	1.69%	1.69%	1.69%	1.88%	1.13%	1.50%									
NiV/BD/HU/2004/FA	JN808858	B	1.88%	1.88%	1.88%	2.26%	1.88%	1.88%	1.88%	2.07%	1.32%	1.69%	0.56%								
NiV/BD/HU/2004/RA2	JN808861	B	1.50%	1.50%	1.50%	1.88%	1.50%	1.50%	1.50%	1.69%	0.94%	1.32%	0.19%	0.38%							
NiV/BD/HU/2004/RAJ	JN808862	B	1.88%	1.88%	1.88%	2.26%	1.88%	1.88%	1.88%	2.07%	1.32%	1.69%	0.56%	0.38%	0.38%						
NiV/BD/HU/2008/MA	JN808857	B	1.50%	1.50%	1.50%	1.88%	1.50%	1.50%	1.50%	1.69%	0.94%	1.32%	0.19%	0.38%	0.00%	0.38%					
NiV/BD/HU/2008/RA	JN808863	B	1.50%	1.50%	1.50%	1.88%	1.50%	1.50%	1.50%	1.69%	0.94%	1.32%	0.19%	0.38%	0.00%	0.38%	0.00%				
NiV/BD/HU/2010/FA1	JN808864	B	1.69%	1.69%	1.69%	2.07%	1.69%	1.69%	1.69%	1.88%	1.13%	1.50%	0.38%	0.56%	0.19%	0.56%	0.19%	0.19%			
NiV/BD/HU/2010/GO	JN808860	B	1.69%	1.69%	1.69%	2.07%	1.69%	1.69%	1.69%	1.88%	1.13%	1.50%	0.38%	0.56%	0.19%	0.56%	0.19%	0.19%	0.38%		
NiV/BD/HU/2010/FA2	JN808859	B	1.50%	1.50%	1.50%	1.88%	1.50%	1.50%	1.50%	1.69%	0.94%	1.32%	0.19%	0.38%	0.00%	0.38%	0.00%	0.00%	0.19%	0.19%	
NiV/IN/HU/2007/FG	FJ513078	B	1.69%	1.69%	1.69%	2.07%	1.69%	1.69%	1.69%	1.88%	1.13%	1.50%	0.38%	0.56%	0.19%	0.56%	0.19%	0.19%	0.38%	0.38%	0.19%

Technical Appendix Figure 1B. Estimates of evolutionary divergence between NiV N ORF amino acid sequences. The number of amino acid differences (top matrix) and percentage of difference (bottom matrix) per sequence from between sequences are shown. SE estimate(s) are shown above the diagonal. The analysis involved 20 aa sequences. Codon positions included were 1st+2nd+3rd+noncoding. There were a total of 532 positions in the final dataset.

Nipah sequence ID	Accession #	Genotype																				
NiV/MY/HU/1999/CDC	AF212302	M																				
NiV/MY/PI/1999/1413	AJ564622	M	0.0																			
NiV/MY/PI/1999/2794	AJ564621	M	0.0	0.0																		
NiV/MY/PI/1999/0128	AJ564623	M	0.0	0.0	0.0																	
NiV/MY/HU/1999/UM1	AY029767	M	0.0	0.0	0.0	0.0																
NiV/MY/HU/1999/UM2	AY029768	M	0.0	0.0	0.0	0.0	0.0															
NiV/MY/BA/2000/TI	AF376747	M	0.0	0.0	0.0	0.0	0.0	0.0														
NiV/MY/PI/1999/0626	AJ627196	M	3.0	3.0	3.0	3.0	3.0	3.0	3.0													
NiV/MY/BA/2010/MY	FN869553	M	3.0	3.0	3.0	3.0	3.0	3.0	3.0	4.0												
NiV/KH/BA/2004/KHM	AY858110	M	14.0	14.0	14.0	14.0	14.0	14.0	14.0	15.0	11.0											
NiV/BD/HU/2004/RA1	AY988601	B	36.0	36.0	36.0	36.0	36.0	36.0	36.0	37.0	35.0	38.0										
NiV/BD/HU/2004/RA2	JN808858	B	37.0	37.0	37.0	37.0	37.0	37.0	37.0	38.0	34.0	37.0	6.0									
NiV/BD/HU/2004/RAJ	JN808861	B	36.0	36.0	36.0	36.0	36.0	36.0	36.0	37.0	35.0	38.0	1.0	5.0								
NiV/BD/HU/2004/FA	JN808862	B	37.0	37.0	37.0	37.0	37.0	37.0	37.0	38.0	36.0	39.0	2.0	6.0	1.0							
NiV/BD/HU/2008/MA	JN808857	B	36.0	36.0	36.0	36.0	36.0	36.0	36.0	37.0	35.0	38.0	5.0	7.0	4.0	5.0						
NiV/BD/HU/2008/RA	JN808863	B	36.0	36.0	36.0	36.0	36.0	36.0	36.0	37.0	35.0	38.0	5.0	7.0	4.0	5.0	0.0					
NiV/BD/HU/2010/FA1	JN808864	B	35.0	35.0	35.0	35.0	35.0	35.0	35.0	36.0	34.0	37.0	2.0	6.0	1.0	2.0	5.0	5.0				
NiV/BD/HU/2010/GO	JN808860	B	37.0	37.0	37.0	37.0	37.0	37.0	37.0	38.0	36.0	39.0	4.0	6.0	3.0	4.0	1.0	1.0	4.0			
NiV/BD/HU/2010/FA2	JN808859	B	35.0	35.0	35.0	35.0	35.0	35.0	35.0	36.0	34.0	39.0	4.0	6.0	3.0	4.0	3.0	3.0	4.0	2.0		
NiV/IN/HU/2007/FG	FJ513078	B	39.0	39.0	39.0	39.0	39.0	39.0	39.0	40.0	38.0	41.0	4.0	8.0	3.0	4.0	3.0	3.0	4.0	2.0	4.0	

Nipah sequence ID	Accession #	Genotype																				
NiV/MY/HU/1999/CDC	AF212302	M																				
NiV/MY/PI/1999/1413	AJ564622	M	0.00%																			
NiV/MY/PI/1999/2794	AJ564621	M	0.00%	0.00%																		
NiV/MY/PI/1999/0128	AJ564623	M	0.00%	0.00%	0.00%																	
NiV/MY/HU/1999/UM1	AY029767	M	0.00%	0.00%	0.00%	0.00%																
NiV/MY/HU/1999/UM2	AY029768	M	0.00%	0.00%	0.00%	0.00%	0.00%															
NiV/MY/BA/2000/TI	AF376747	M	0.00%	0.00%	0.00%	0.00%	0.00%	0.00%														
NiV/MY/PI/1999/0626	AJ627196	M	0.41%	0.41%	0.41%	0.41%	0.41%	0.41%	0.41%													
NiV/MY/BA/2010/MY	FN869553	M	0.41%	0.41%	0.41%	0.41%	0.41%	0.41%	0.41%	0.55%												
NiV/KH/BA/2004/KHM	AY858110	M	1.92%	1.92%	1.92%	1.92%	1.92%	1.92%	1.92%	2.06%	1.51%											
NiV/BD/HU/2004/RA1	AY988601	B	4.94%	4.94%	4.94%	4.94%	4.94%	4.94%	4.94%	5.08%	4.80%	5.21%										
NiV/BD/HU/2004/RA2	JN808858	B	5.08%	5.08%	5.08%	5.08%	5.08%	5.08%	5.08%	5.21%	4.66%	5.08%	0.82%									
NiV/BD/HU/2004/RAJ	JN808861	B	4.94%	4.94%	4.94%	4.94%	4.94%	4.94%	4.94%	5.08%	4.80%	5.21%	0.14%	0.69%								
NiV/BD/HU/2004/FA	JN808862	B	5.08%	5.08%	5.08%	5.08%	5.08%	5.08%	5.08%	5.21%	4.94%	5.35%	0.27%	0.82%	0.14%							
NiV/BD/HU/2008/MA	JN808857	B	4.94%	4.94%	4.94%	4.94%	4.94%	4.94%	4.94%	5.08%	4.80%	5.21%	0.69%	0.96%	0.55%	0.69%						
NiV/BD/HU/2008/RA	JN808863	B	4.94%	4.94%	4.94%	4.94%	4.94%	4.94%	4.94%	5.08%	4.80%	5.21%	0.69%	0.96%	0.55%	0.69%	0.00%					
NiV/BD/HU/2010/FA1	JN808864	B	4.80%	4.80%	4.80%	4.80%	4.80%	4.80%	4.80%	4.94%	4.66%	5.08%	0.27%	0.82%	0.14%	0.27%	0.69%	0.69%				
NiV/BD/HU/2010/GO	JN808860	B	5.08%	5.08%	5.08%	5.08%	5.08%	5.08%	5.08%	5.21%	4.94%	5.35%	0.55%	0.82%	0.41%	0.55%	0.14%	0.14%	0.55%			
NiV/BD/HU/2010/FA2	JN808859	B	4.80%	4.80%	4.80%	4.80%	4.80%	4.80%	4.80%	4.94%	4.66%	5.35%	0.55%	0.82%	0.41%	0.55%	0.41%	0.41%	0.55%	0.27%		
NiV/IN/HU/2007/FG	FJ513078	B	5.35%	5.35%	5.35%	5.35%	5.35%	5.35%	5.35%	5.49%	5.21%	5.62%	0.55%	1.10%	0.41%	0.55%	0.41%	0.41%	0.55%	0.27%	0.55%	

Technical Appendix Figure 1C. Estimates of evolutionary divergence between NiV N ORF nucleotide sequences within a 729-nt window. The number of base differences (top matrix) and percentage of differences (bottom matrix) per sequence from between sequences are shown. SE estimate(s) are shown above the diagonal. The analysis involved 20 nt sequences. Codon positions included were 1st+2nd+3rd+noncoding. There were a total of 729 positions in the final dataset.

Nipah sequence ID	Accession #	Genotype																			
NiV/MY/HU/1999/CDC	AF212302	M																			
NiV/MY/PI/1999/1413	AJ564622	M	0.0																		
NiV/MY/PI/1999/2794	AJ564621	M	0.0	0.0																	
NiV/MY/HU/1999/0128	AJ564623	M	0.0	0.0	0.0																
NiV/MY/HU/1999/UM1	AY029767	M	0.0	0.0	0.0	0.0															
NiV/MY/HU/1999/UM2	AY029768	M	0.0	0.0	0.0	0.0	0.0														
NiV/MY/BA/2000/TI	AF376747	M	0.0	0.0	0.0	0.0	0.0	0.0													
NiV/MY/PI/1999/0626	AJ627196	M	0.0	0.0	0.0	0.0	0.0	0.0	0.0												
NiV/MY/BA/2010/MY	FN869553	M	7.0	7.0	7.0	7.0	7.0	7.0	7.0	7.0											
NiV/KH/BA/2004/KHM	AY858110	M	6.0	6.0	6.0	6.0	6.0	6.0	6.0	6.0	5.0										
NiV/BD/HU/2004/RA1	AY988601	B	24.0	24.0	24.0	24.0	24.0	24.0	24.0	24.0	23.0	22.0									
NiV/BD/HU/2004/FA	JN808858	B	27.0	27.0	27.0	27.0	27.0	27.0	27.0	27.0	26.0	25.0	3.0								
NiV/BD/HU/2004/RA2	JN808861	B	23.0	23.0	23.0	23.0	23.0	23.0	23.0	23.0	22.0	21.0	3.0	6.0							
NiV/BD/HU/2004/RAJ	JN808862	B	28.0	28.0	28.0	28.0	28.0	28.0	28.0	28.0	27.0	26.0	4.0	3.0	7.0						
NiV/BD/HU/2008/MA	JN808857	B	25.0	25.0	25.0	25.0	25.0	25.0	25.0	25.0	24.0	23.0	3.0	6.0	4.0	7.0					
NiV/BD/HU/2008/RA	JN808863	B	25.0	25.0	25.0	25.0	25.0	25.0	25.0	25.0	24.0	23.0	3.0	6.0	4.0	7.0	0.0				
NiV/BD/HU/2010/FA1	JN808864	B	25.0	25.0	25.0	25.0	25.0	25.0	25.0	25.0	24.0	23.0	1.0	4.0	4.0	5.0	4.0	4.0			
NiV/BD/HU/2010/GO	JN808860	B	25.0	25.0	25.0	25.0	25.0	25.0	25.0	25.0	24.0	23.0	3.0	6.0	4.0	7.0	2.0	2.0	4.0		
NiV/BD/HU/2010/FA2	JN808859	B	25.0	25.0	25.0	25.0	25.0	25.0	25.0	25.0	24.0	23.0	3.0	6.0	4.0	7.0	2.0	2.0	4.0	2.0	
NiV/IN/HU/2007/FG	FJ513078	B	24.0	24.0	24.0	24.0	24.0	24.0	24.0	24.0	23.0	22.0	2.0	5.0	3.0	6.0	1.0	1.0	3.0	1.0	1.0

Nipah sequence ID	Accession #	Genotype																			
NiV/MY/HU/1999/CDC	AF212302	M																			
NiV/MY/PI/1999/1413	AJ564622	M	0.00%																		
NiV/MY/PI/1999/2794	AJ564621	M	0.00%	0.00%																	
NiV/MY/HU/1999/0128	AJ564623	M	0.00%	0.00%	0.00%																
NiV/MY/HU/1999/UM1	AY029767	M	0.00%	0.00%	0.00%	0.00%															
NiV/MY/HU/1999/UM2	AY029768	M	0.00%	0.00%	0.00%	0.00%	0.00%														
NiV/MY/BA/2000/TI	AF376747	M	0.00%	0.00%	0.00%	0.00%	0.00%	0.00%													
NiV/MY/PI/1999/0626	AJ627196	M	0.00%	0.00%	0.00%	0.00%	0.00%	0.00%	0.00%												
NiV/MY/BA/2010/MY	FN869553	M	1.96%	1.96%	1.96%	1.96%	1.96%	1.96%	1.96%	1.96%											
NiV/KH/BA/2004/KHM	AY858110	M	1.68%	1.68%	1.68%	1.68%	1.68%	1.68%	1.68%	1.68%	1.40%										
NiV/BD/HU/2004/RA1	AY988601	B	6.72%	6.72%	6.72%	6.72%	6.72%	6.72%	6.72%	6.72%	6.44%	6.16%									
NiV/BD/HU/2004/FA	JN808858	B	7.56%	7.56%	7.56%	7.56%	7.56%	7.56%	7.56%	7.56%	7.28%	7.00%	0.84%								
NiV/BD/HU/2004/RA2	JN808861	B	6.44%	6.44%	6.44%	6.44%	6.44%	6.44%	6.44%	6.44%	6.16%	5.88%	0.84%	1.68%							
NiV/BD/HU/2004/RAJ	JN808862	B	7.84%	7.84%	7.84%	7.84%	7.84%	7.84%	7.84%	7.84%	7.56%	7.28%	1.12%	0.84%	1.96%						
NiV/BD/HU/2008/MA	JN808857	B	7.00%	7.00%	7.00%	7.00%	7.00%	7.00%	7.00%	7.00%	6.72%	6.44%	0.84%	1.68%	1.12%	1.96%					
NiV/BD/HU/2008/RA	JN808863	B	7.00%	7.00%	7.00%	7.00%	7.00%	7.00%	7.00%	7.00%	6.72%	6.44%	0.84%	1.68%	1.12%	1.96%	0.00%				
NiV/BD/HU/2010/FA1	JN808864	B	7.00%	7.00%	7.00%	7.00%	7.00%	7.00%	7.00%	7.00%	6.72%	6.44%	0.28%	1.12%	1.12%	1.40%	1.12%	1.12%			
NiV/BD/HU/2010/GO	JN808860	B	7.00%	7.00%	7.00%	7.00%	7.00%	7.00%	7.00%	7.00%	6.72%	6.44%	0.84%	1.68%	1.12%	1.96%	0.56%	0.56%	1.12%		
NiV/BD/HU/2010/FA2	JN808859	B	7.00%	7.00%	7.00%	7.00%	7.00%	7.00%	7.00%	7.00%	6.72%	6.44%	0.84%	1.68%	1.12%	1.96%	0.56%	0.56%	1.12%	0.56%	
NiV/IN/HU/2007/FG	FJ513078	B	6.72%	6.72%	6.72%	6.72%	6.72%	6.72%	6.72%	6.72%	6.44%	6.16%	0.56%	1.40%	0.84%	1.68%	0.28%	0.28%	0.84%	0.28%	0.28%

Technical Appendix Figure 1D. Estimates of evolutionary divergence between NiV N ORF nucleotide sequences within a 357-nt window. The number of base differences (top matrix) and percentage of differences (bottom matrix) per sequence from between sequences are shown. SE estimate(s) are shown above the diagonal. The analysis involved 20 nt sequences. Codon positions included were 1st+2nd+3rd+noncoding. There were a total of 357 positions in the final dataset.

Nipah sequence ID	Accession #	Genotype													
NiV/MY/HU/1999/CDC	AF212302	M													
NiV/MY/PI/1999/1413	AJ564622	M	0.0												
NiV/MY/PI/1999/2794	AJ564621	M	0.0	0.0											
NiV/MY/HU/1999/0128	AJ564623	M	0.0	0.0	0.0										
NiV/MY/HU/1999/UM1	AY029767	M	0.0	0.0	0.0	0.0									
NiV/MY/HU/1999/UM2	AY029768	M	0.0	0.0	0.0	0.0	0.0								
NiV/MY/BA/2000/TI	AF376747	M	1.0	1.0	1.0	1.0	1.0	1.0							
NiV/MY/PI/1999/0626	AJ627196	M	5.0	5.0	5.0	5.0	5.0	5.0	6.0						
NiV/MY/BA/2010/MY	FN869553	M	46.0	46.0	46.0	46.0	46.0	46.0	47.0	47.0					
NiV/BD/HU/2004/RA1	AY988601	B	171.0	171.0	171.0	171.0	171.0	171.0	172.0	172.0	186.0				
NiV/BD/HU/2008/MA	JN808857	B	179.0	179.0	179.0	179.0	179.0	179.0	180.0	180.0	194.0	14.0			
NiV/BD/HU/2008/RA	JN808863	B	179.0	179.0	179.0	179.0	179.0	179.0	180.0	180.0	194.0	14.0	0.0		
NiV/BD/HU/2010/FA1	JN808864	B	180.0	180.0	180.0	180.0	180.0	180.0	181.0	181.0	195.0	9.0	21.0	21.0	
NiV/IN/HU/2007/FG	FJ513078	B	177.0	177.0	177.0	177.0	177.0	177.0	178.0	178.0	193.0	11.0	11.0	11.0	20.0

Nipah sequence ID	Accession #	Genotype													
NiV/MY/HU/1999/CDC	AF212302	M													
NiV/MY/PI/1999/1413	AJ564622	M	0.00%												
NiV/MY/PI/1999/2794	AJ564621	M	0.00%	0.00%											
NiV/MY/HU/1999/0128	AJ564623	M	0.00%	0.00%	0.00%										
NiV/MY/HU/1999/UM1	AY029767	M	0.00%	0.00%	0.00%	0.00%									
NiV/MY/HU/1999/UM2	AY029768	M	0.00%	0.00%	0.00%	0.00%	0.00%								
NiV/MY/BA/2000/TI	AF376747	M	0.05%	0.05%	0.05%	0.05%	0.05%	0.05%							
NiV/MY/PI/1999/0626	AJ627196	M	0.23%	0.23%	0.23%	0.23%	0.23%	0.23%	0.28%						
NiV/MY/BA/2010/MY	FN869553	M	2.16%	2.16%	2.16%	2.16%	2.16%	2.16%	2.21%	2.21%					
NiV/BD/HU/2004/RA1	AY988601	B	8.03%	8.03%	8.03%	8.03%	8.03%	8.03%	8.08%	8.08%	8.73%				
NiV/BD/HU/2008/MA	JN808857	B	8.40%	8.40%	8.40%	8.40%	8.40%	8.40%	8.45%	8.45%	9.11%	0.66%			
NiV/BD/HU/2008/RA	JN808863	B	8.40%	8.40%	8.40%	8.40%	8.40%	8.40%	8.45%	8.45%	9.11%	0.66%	0.00%		
NiV/BD/HU/2010/FA1	JN808864	B	8.45%	8.45%	8.45%	8.45%	8.45%	8.45%	8.50%	8.50%	9.15%	0.42%	0.99%	0.99%	
NiV/IN/HU/2007/FG	FJ513078	B	8.31%	8.31%	8.31%	8.31%	8.31%	8.31%	8.36%	8.36%	9.06%	0.52%	0.52%	0.52%	0.94%

Technical Appendix Figure 2A. Estimates of evolutionary divergence between NiV P ORF nucleotide sequences. The number of base differences (top matrix) and percentage of difference (bottom matrix) per sequence from between sequences are shown. SE estimate(s) are shown above the diagonal. The analysis involved 14 nt sequences. Codon positions included were 1st+2nd+3rd+noncoding. There were a total of 2,130 positions in the final dataset.

Nipah sequence ID	Accession #	Genotype													
NiV/MY/HU/1999/CDC	AF212302	M													
NiV/MY/PI/1999/1413	AJ564622	M	0.0												
NiV/MY/PI/1999/2794	AJ564621	M	0.0	0.0											
NiV/MY/HU/1999/0128	AJ564623	M	0.0	0.0	0.0										
NiV/MY/HU/1999/UM1	AY029767	M	0.0	0.0	0.0	0.0									
NiV/MY/HU/1999/UM2	AY029768	M	0.0	0.0	0.0	0.0	0.0								
NiV/MY/BA/2000/TI	AF376747	M	1.0	1.0	1.0	1.0	1.0	1.0							
NiV/MY/PI/1999/0626	AJ627196	M	3.0	3.0	3.0	3.0	3.0	3.0	4.0						
NiV/MY/BA/2010/MY	FN869553	M	25.0	25.0	25.0	25.0	25.0	25.0	26.0	26.0					
NiV/BD/HU/2004/RA1	AY988601	B	57.0	57.0	57.0	57.0	57.0	57.0	58.0	58.0	68.0				
NiV/BD/HU/2008/MA	JN808857	B	59.0	59.0	59.0	59.0	59.0	59.0	60.0	60.0	70.0	5.0			
NiV/BD/HU/2008/RA	JN808863	B	59.0	59.0	59.0	59.0	59.0	59.0	60.0	60.0	70.0	5.0	0.0		
NiV/BD/HU/2010/FA1	JN808864	B	59.0	59.0	59.0	59.0	59.0	59.0	60.0	60.0	70.0	2.0	7.0	7.0	
NiV/IN/HU/2007/FG	FJ513078	B	59.0	59.0	59.0	59.0	59.0	59.0	60.0	60.0	70.0	4.0	5.0	5.0	6.0

Nipah sequence ID	Accession #	Genotype													
NiV/MY/HU/1999/CDC	AF212302	M													
NiV/MY/PI/1999/1413	AJ564622	M	0.00%												
NiV/MY/PI/1999/2794	AJ564621	M	0.00%	0.00%											
NiV/MY/HU/1999/0128	AJ564623	M	0.00%	0.00%	0.00%										
NiV/MY/HU/1999/UM1	AY029767	M	0.00%	0.00%	0.00%	0.00%									
NiV/MY/HU/1999/UM2	AY029768	M	0.00%	0.00%	0.00%	0.00%	0.00%								
NiV/MY/BA/2000/TI	AF376747	M	0.14%	0.14%	0.14%	0.14%	0.14%	0.14%							
NiV/MY/PI/1999/0626	AJ627196	M	0.42%	0.42%	0.42%	0.42%	0.42%	0.42%	0.56%						
NiV/MY/BA/2010/MY	FN869553	M	3.53%	3.53%	3.53%	3.53%	3.53%	3.53%	3.67%	3.67%					
NiV/BD/HU/2004/RA1	AY988601	B	8.04%	8.04%	8.04%	8.04%	8.04%	8.04%	8.18%	8.18%	9.59%				
NiV/BD/HU/2008/MA	JN808857	B	8.32%	8.32%	8.32%	8.32%	8.32%	8.32%	8.46%	8.46%	9.87%	0.71%			
NiV/BD/HU/2008/RA	JN808863	B	8.32%	8.32%	8.32%	8.32%	8.32%	8.32%	8.46%	8.46%	9.87%	0.71%	0.00%		
NiV/BD/HU/2010/FA1	JN808864	B	8.32%	8.32%	8.32%	8.32%	8.32%	8.32%	8.46%	8.46%	9.87%	0.28%	0.99%	0.99%	
NiV/IN/HU/2007/FG	FJ513078	B	8.32%	8.32%	8.32%	8.32%	8.32%	8.32%	8.46%	8.46%	9.87%	0.56%	0.71%	0.71%	0.85%

Technical Appendix Figure 2B. Estimates of evolutionary divergence between NiV P ORF amino acid sequences. The number of amino acid differences (top matrix) and percentage of difference (bottom matrix) per sequence from between sequences are shown. SE estimate(s) are shown above the diagonal. The analysis involved 14 aa sequences. Codon positions included were 1st+2nd+3rd+noncoding. There were a total of 709 positions in the final dataset.

Nipah sequence ID	Accession #	Genotype													
NiV/MY/HU/1999/CDC	AF212302	M													
NiV/MY/PI/1999/1413	AJ564622	M	0.00												
NiV/MY/PI/1999/2794	AJ564621	M	0.00	0.00											
NiV/MY/HU/1999/0128	AJ564623	M	0.00	0.00	0.00										
NiV/MY/HU/1999/UM1	AY029767	M	0.00	0.00	0.00	0.00									
NiV/MY/HU/1999/UM2	AY029768	M	0.00	0.00	0.00	0.00	0.00								
NiV/MY/BA/2000/TI	AF376747	M	0.00	0.00	0.00	0.00	0.00	0.00							
NiV/MY/PI/1999/0626	AJ627196	M	3.00	3.00	3.00	3.00	3.00	3.00	3.00						
NiV/MY/BA/2010/MY	FN869553	M	6.00	6.00	6.00	6.00	6.00	6.00	6.00	5.00					
NiV/BD/HU/2004/RA1	AY988601	B	70.00	70.00	70.00	70.00	70.00	70.00	70.00	69.00	66.00				
NiV/BD/HU/2008/MA	JN808857	B	70.00	70.00	70.00	70.00	70.00	70.00	70.00	69.00	66.00	2.00			
NiV/BD/HU/2008/RA	JN808863	B	70.00	70.00	70.00	70.00	70.00	70.00	70.00	69.00	66.00	2.00	0.00		
NiV/BD/HU/2010/FA1	JN808864	B	70.00	70.00	70.00	70.00	70.00	70.00	70.00	69.00	66.00	0.00	2.00	2.00	
NiV/IN/HU/2007/FG	FJ513078	B	71.00	71.00	71.00	71.00	71.00	71.00	71.00	70.00	67.00	3.00	1.00	1.00	3.00

Nipah sequence ID	Accession #	Genotype													
NiV/MY/HU/1999/CDC	AF212302	M													
NiV/MY/PI/1999/1413	AJ564622	M	0.00%												
NiV/MY/PI/1999/2794	AJ564621	M	0.00%	0.00%											
NiV/MY/HU/1999/0128	AJ564623	M	0.00%	0.00%	0.00%										
NiV/MY/HU/1999/UM1	AY029767	M	0.00%	0.00%	0.00%	0.00%									
NiV/MY/HU/1999/UM2	AY029768	M	0.00%	0.00%	0.00%	0.00%	0.00%								
NiV/MY/BA/2000/TI	AF376747	M	0.00%	0.00%	0.00%	0.00%	0.00%	0.00%							
NiV/MY/PI/1999/0626	AJ627196	M	0.28%	0.28%	0.28%	0.28%	0.28%	0.28%	0.28%						
NiV/MY/BA/2010/MY	FN869553	M	0.57%	0.57%	0.57%	0.57%	0.57%	0.57%	0.57%	0.47%					
NiV/BD/HU/2004/RA1	AY988601	B	6.61%	6.61%	6.61%	6.61%	6.61%	6.61%	6.61%	6.52%	6.23%				
NiV/BD/HU/2008/MA	JN808857	B	6.61%	6.61%	6.61%	6.61%	6.61%	6.61%	6.61%	6.52%	6.23%	0.19%			
NiV/BD/HU/2008/RA	JN808863	B	6.61%	6.61%	6.61%	6.61%	6.61%	6.61%	6.61%	6.52%	6.23%	0.19%	0.00%		
NiV/BD/HU/2010/FA1	JN808864	B	6.61%	6.61%	6.61%	6.61%	6.61%	6.61%	6.61%	6.52%	6.23%	0.00%	0.19%	0.19%	
NiV/IN/HU/2007/FG	FJ513078	B	6.70%	6.70%	6.70%	6.70%	6.70%	6.70%	6.70%	6.61%	6.33%	0.28%	0.09%	0.09%	0.28%

Technical Appendix Figure 3A. Estimates of evolutionary divergence between NiV M ORF nucleotide sequences. The number of base differences (top matrix) and percentage of difference (bottom matrix) per sequence from between sequences are shown. SE estimate(s) are shown above the diagonal. The analysis involved 14 nt sequences. Codon positions included were 1st+2nd+3rd+noncoding. There were a total of 1,059 positions in the final dataset.

Nipah sequence ID	Accession #	Genotype													
NiV/MY/HU/1999/CDC	AF212302	M													
NiV/MY/PI/1999/1413	AJ564622	M	0.0												
NiV/MY/PI/1999/2794	AJ564621	M	0.0	0.0											
NiV/MY/HU/1999/0128	AJ564623	M	0.0	0.0	0.0										
NiV/MY/HU/1999/UM1	AY029767	M	0.0	0.0	0.0	0.0									
NiV/MY/HU/1999/UM2	AY029768	M	0.0	0.0	0.0	0.0	0.0								
NiV/MY/BA/2000/TI	AF376747	M	0.0	0.0	0.0	0.0	0.0	0.0							
NiV/MY/PI/1999/0626	AJ627196	M	1.0	1.0	1.0	1.0	1.0	1.0	1.0						
NiV/MY/BA/2010/MY	FN869553	M	3.0	3.0	3.0	3.0	3.0	3.0	3.0	2.0					
NiV/BD/HU/2004/RA1	AY988601	B	4.0	4.0	4.0	4.0	4.0	4.0	4.0	3.0	3.0				
NiV/BD/HU/2008/MA	JN808857	B	4.0	4.0	4.0	4.0	4.0	4.0	4.0	3.0	3.0	0.0			
NiV/BD/HU/2008/RA	JN808863	B	4.0	4.0	4.0	4.0	4.0	4.0	4.0	3.0	3.0	0.0	0.0		
NiV/BD/HU/2010/FA1	JN808864	B	4.0	4.0	4.0	4.0	4.0	4.0	4.0	3.0	3.0	0.0	0.0	0.0	
NiV/IN/HU/2007/FG	FJ513078	B	5.0	5.0	5.0	5.0	5.0	5.0	5.0	4.0	4.0	1.0	1.0	1.0	1.0

Nipah sequence ID	Accession #	Genotype													
NiV/MY/HU/1999/CDC	AF212302	M													
NiV/MY/PI/1999/1413	AJ564622	M	0.00%												
NiV/MY/PI/1999/2794	AJ564621	M	0.00%	0.00%											
NiV/MY/HU/1999/0128	AJ564623	M	0.00%	0.00%	0.00%										
NiV/MY/HU/1999/UM1	AY029767	M	0.00%	0.00%	0.00%	0.00%									
NiV/MY/HU/1999/UM2	AY029768	M	0.00%	0.00%	0.00%	0.00%	0.00%								
NiV/MY/BA/2000/TI	AF376747	M	0.00%	0.00%	0.00%	0.00%	0.00%	0.00%							
NiV/MY/PI/1999/0626	AJ627196	M	0.28%	0.28%	0.28%	0.28%	0.28%	0.28%	0.28%						
NiV/MY/BA/2010/MY	FN869553	M	0.85%	0.85%	0.85%	0.85%	0.85%	0.85%	0.85%	0.57%					
NiV/BD/HU/2004/RA1	AY988601	B	1.14%	1.14%	1.14%	1.14%	1.14%	1.14%	1.14%	0.85%	0.85%				
NiV/BD/HU/2008/MA	JN808857	B	1.14%	1.14%	1.14%	1.14%	1.14%	1.14%	1.14%	0.85%	0.85%	0.00%			
NiV/BD/HU/2008/RA	JN808863	B	1.14%	1.14%	1.14%	1.14%	1.14%	1.14%	1.14%	0.85%	0.85%	0.00%	0.00%		
NiV/BD/HU/2010/FA1	JN808864	B	1.14%	1.14%	1.14%	1.14%	1.14%	1.14%	1.14%	0.85%	0.85%	0.00%	0.00%	0.00%	
NiV/IN/HU/2007/FG	FJ513078	B	1.42%	1.42%	1.42%	1.42%	1.42%	1.42%	1.42%	1.14%	1.14%	0.28%	0.28%	0.28%	0.28%

Technical Appendix Figure 3B. Estimates of evolutionary divergence between NiV M ORF amino acid sequences. The number of amino acid differences (top matrix) and percentage of difference (bottom matrix) per sequence from between sequences are shown. SE estimate(s) are shown above the diagonal. The analysis involved 14 aa sequences. Codon positions included were 1st+2nd+3rd+noncoding. There were a total of 352 positions in the final dataset.

Nipah sequence ID	Accession #	Genotype													
NiV/MY/HU/1999/CDC	AF212302	M													
NiV/MY/PI/1999/1413	AJ564622	M	0.00												
NiV/MY/PI/1999/2794	AJ564621	M	1.00	1.00											
NiV/MY/HU/1999/0128	AJ564623	M	0.00	0.00	1.00										
NiV/MY/HU/1999/UM1	AY029767	M	0.00	0.00	1.00	0.00									
NiV/MY/HU/1999/UM2	AY029768	M	0.00	0.00	1.00	0.00	0.00								
NiV/MY/BA/2000/TI	AF376747	M	1.00	1.00	2.00	1.00	1.00	1.00							
NiV/MY/PI/1999/0626	AJ627196	M	4.00	4.00	5.00	4.00	4.00	4.00	5.00						
NiV/MY/BA/2010/MY	FN869553	M	10.00	10.00	11.00	10.00	10.00	10.00	11.00	14.00					
NiV/BD/HU/2004/RA1	AY988601	B	107.00	107.00	108.00	107.00	107.00	107.00	108.00	109.00	107.00				
NiV/BD/HU/2008/MA	JN808857	B	109.00	109.00	110.00	109.00	109.00	109.00	110.00	111.00	109.00	16.00			
NiV/BD/HU/2008/RA	JN808863	B	109.00	109.00	110.00	109.00	109.00	109.00	110.00	111.00	109.00	16.00	0.00		
NiV/BD/HU/2010/FA1	JN808864	B	105.00	105.00	106.00	105.00	105.00	105.00	106.00	107.00	105.00	8.00	14.00	14.00	
NiV/IN/HU/2007/FG	FJ513078	B	106.00	106.00	107.00	106.00	106.00	106.00	107.00	108.00	106.00	11.00	5.00	5.00	9.00

Nipah sequence ID	Accession #	Genotype													
NiV/MY/HU/1999/CDC	AF212302	M													
NiV/MY/PI/1999/1413	AJ564622	M	0.00%												
NiV/MY/PI/1999/2794	AJ564621	M	0.06%	0.06%											
NiV/MY/HU/1999/0128	AJ564623	M	0.00%	0.00%	0.06%										
NiV/MY/HU/1999/UM1	AY029767	M	0.00%	0.00%	0.06%	0.00%									
NiV/MY/HU/1999/UM2	AY029768	M	0.00%	0.00%	0.06%	0.00%	0.00%								
NiV/MY/BA/2000/TI	AF376747	M	0.06%	0.06%	0.12%	0.06%	0.06%	0.06%							
NiV/MY/PI/1999/0626	AJ627196	M	0.24%	0.24%	0.30%	0.24%	0.24%	0.24%	0.30%						
NiV/MY/BA/2010/MY	FN869553	M	0.61%	0.61%	0.67%	0.61%	0.61%	0.61%	0.67%	0.85%					
NiV/BD/HU/2004/RA1	AY988601	B	6.52%	6.52%	6.58%	6.52%	6.52%	6.52%	6.58%	6.64%	6.52%				
NiV/BD/HU/2008/MA	JN808857	B	6.64%	6.64%	6.70%	6.64%	6.64%	6.64%	6.70%	6.76%	6.64%	0.98%			
NiV/BD/HU/2008/RA	JN808863	B	6.64%	6.64%	6.70%	6.64%	6.64%	6.64%	6.70%	6.76%	6.64%	0.98%	0.00%		
NiV/BD/HU/2010/FA1	JN808864	B	6.40%	6.40%	6.46%	6.40%	6.40%	6.40%	6.46%	6.52%	6.40%	0.49%	0.85%	0.85%	
NiV/IN/HU/2007/FG	FJ513078	B	6.46%	6.46%	6.52%	6.46%	6.46%	6.46%	6.52%	6.58%	6.46%	0.67%	0.30%	0.30%	0.55%

Technical Appendix Figure 4A. Estimates of evolutionary divergence between NiV F ORF nucleotide sequences. The number of base differences (top matrix) and percentage of difference (bottom matrix) per sequence from between sequences are shown. SE estimate(s) are shown above the diagonal. The analysis involved 14 nt sequences. Codon positions included were 1st+2nd+3rd+noncoding. There were a total of 1,641 positions in the final dataset.

Nipah sequence ID	Accession #	Genotype													
NiV/MY/HU/1999/CDC	AF212302	M													
NiV/MY/PI/1999/1413	AJ564622	M	0.00												
NiV/MY/PI/1999/2794	AJ564621	M	0.00	0.00											
NiV/MY/HU/1999/0128	AJ564623	M	0.00	0.00	0.00										
NiV/MY/HU/1999/UM1	AY029767	M	0.00	0.00	0.00	0.00									
NiV/MY/HU/1999/UM2	AY029768	M	0.00	0.00	0.00	0.00	0.00								
NiV/MY/BA/2000/TI	AF376747	M	1.00	1.00	1.00	1.00	1.00	1.00							
NiV/MY/PI/1999/0626	AJ627196	M	1.00	1.00	1.00	1.00	1.00	1.00	2.00						
NiV/MY/BA/2010/MY	FN869553	M	3.00	3.00	3.00	3.00	3.00	3.00	4.00	4.00					
NiV/BD/HU/2004/RA1	AY988601	B	8.00	8.00	8.00	8.00	8.00	8.00	9.00	9.00	9.00				
NiV/BD/HU/2008/MA	JN808857	B	7.00	7.00	7.00	7.00	7.00	7.00	8.00	8.00	8.00	3.00			
NiV/BD/HU/2008/RA	JN808863	B	7.00	7.00	7.00	7.00	7.00	7.00	8.00	8.00	8.00	3.00	0.00		
NiV/BD/HU/2010/FA1	JN808864	B	6.00	6.00	6.00	6.00	6.00	6.00	7.00	7.00	7.00	2.00	1.00	1.00	
NiV/IN/HU/2007/FG	FJ513078	B	7.00	7.00	7.00	7.00	7.00	7.00	8.00	8.00	8.00	3.00	0.00	0.00	1.00

Nipah sequence ID	Accession #	Genotype													
NiV/MY/HU/1999/CDC	AF212302	M													
NiV/MY/PI/1999/1413	AJ564622	M	0.00%												
NiV/MY/PI/1999/2794	AJ564621	M	0.00%	0.00%											
NiV/MY/HU/1999/0128	AJ564623	M	0.00%	0.00%	0.00%										
NiV/MY/HU/1999/UM1	AY029767	M	0.00%	0.00%	0.00%	0.00%									
NiV/MY/HU/1999/UM2	AY029768	M	0.00%	0.00%	0.00%	0.00%	0.00%								
NiV/MY/BA/2000/TI	AF376747	M	0.18%	0.18%	0.18%	0.18%	0.18%	0.18%							
NiV/MY/PI/1999/0626	AJ627196	M	0.18%	0.18%	0.18%	0.18%	0.18%	0.18%	0.37%						
NiV/MY/BA/2010/MY	FN869553	M	0.55%	0.55%	0.55%	0.55%	0.55%	0.55%	0.73%	0.73%					
NiV/BD/HU/2004/RA1	AY988601	B	1.47%	1.47%	1.47%	1.47%	1.47%	1.47%	1.65%	1.65%	1.65%				
NiV/BD/HU/2008/MA	JN808857	B	1.28%	1.28%	1.28%	1.28%	1.28%	1.28%	1.47%	1.47%	1.47%	0.55%			
NiV/BD/HU/2008/RA	JN808863	B	1.28%	1.28%	1.28%	1.28%	1.28%	1.28%	1.47%	1.47%	1.47%	0.55%	0.00%		
NiV/BD/HU/2010/FA1	JN808864	B	1.10%	1.10%	1.10%	1.10%	1.10%	1.10%	1.28%	1.28%	1.28%	0.37%	0.18%	0.18%	
NiV/IN/HU/2007/FG	FJ513078	B	1.28%	1.28%	1.28%	1.28%	1.28%	1.28%	1.47%	1.47%	1.47%	0.55%	0.00%	0.00%	0.18%

Technical Appendix Figure 4B. Estimates of evolutionary divergence between NiV F ORF amino acid sequences. The number of amino acid differences (top matrix) and percentage of difference (bottom matrix) per sequence from between sequences are shown. SE estimate(s) are shown above the diagonal. The analysis involved 14 aa sequences. Codon positions included were 1st+2nd+3rd+noncoding. There were a total of 546 positions in the final dataset.

Nipah sequence ID	Accession #	Genotype														
NiV/MY/HU/1999/CDC	AF212302	M														
NiV/MY/PI/1999/1413	AJ564622	M	0.0													
NiV/MY/PI/1999/2794	AJ564621	M	1.0	1.0												
NiV/MY/HU/1999/0128	AJ564623	M	1.0	1.0	0.0											
NiV/MY/HU/1999/UM1	AY029767	M	0.0	0.0	1.0	1.0										
NiV/MY/HU/1999/UM2	AY029768	M	0.0	0.0	1.0	1.0	0.0									
NiV/MY/BA/2000/TI	AF376747	M	2.0	2.0	1.0	1.0	2.0	2.0								
NiV/MY/PI/1999/0626	AJ627196	M	4.0	4.0	5.0	5.0	4.0	4.0	6.0							
NiV/MY/BA/2010/MY	FN869553	M	13.0	13.0	14.0	14.0	13.0	13.0	15.0	13.0						
NiV/KH/BA/2004/KHM	AY858111	M	33.0	33.0	34.0	34.0	33.0	33.0	35.0	35.0	32.0					
NiV/BD/HU/2004/RA1	AY988601	B	126.0	126.0	127.0	127.0	126.0	126.0	128.0	126.0	122.0	121.0				
NiV/BD/HU/2008/MA	JN808857	B	131.0	131.0	132.0	132.0	131.0	131.0	133.0	131.0	127.0	126.0	7.0			
NiV/BD/HU/2008/RA	JN808863	B	131.0	131.0	132.0	132.0	131.0	131.0	133.0	131.0	127.0	126.0	7.0	0.0		
NiV/BD/HU/2010/FA1	JN808864	B	128.0	128.0	129.0	129.0	128.0	128.0	130.0	128.0	124.0	123.0	3.0	10.0	10.0	
NiV/IN/HU/2007/FG	FJ513078	B	129.0	129.0	130.0	130.0	129.0	129.0	131.0	129.0	125.0	124.0	7.0	8.0	8.0	10.0

Nipah sequence ID	Accession #	Genotype														
NiV/MY/HU/1999/CDC	AF212302	M														
NiV/MY/PI/1999/1413	AJ564622	M	0.00%													
NiV/MY/PI/1999/2794	AJ564621	M	0.06%	0.06%												
NiV/MY/HU/1999/0128	AJ564623	M	0.06%	0.06%	0.00%											
NiV/MY/HU/1999/UM1	AY029767	M	0.00%	0.00%	0.06%	0.06%										
NiV/MY/HU/1999/UM2	AY029768	M	0.00%	0.00%	0.06%	0.06%	0.00%									
NiV/MY/BA/2000/TI	AF376747	M	0.11%	0.11%	0.06%	0.06%	0.11%	0.11%								
NiV/MY/PI/1999/0626	AJ627196	M	0.22%	0.22%	0.28%	0.28%	0.22%	0.22%	0.33%							
NiV/MY/BA/2010/MY	FN869553	M	0.72%	0.72%	0.77%	0.77%	0.72%	0.72%	0.83%	0.72%						
NiV/KH/BA/2004/KHM	AY858111	M	1.82%	1.82%	1.88%	1.88%	1.82%	1.82%	1.93%	1.93%	1.77%					
NiV/BD/HU/2004/RA1	AY988601	B	6.97%	6.97%	7.02%	7.02%	6.97%	6.97%	7.08%	6.97%	6.74%	6.69%				
NiV/BD/HU/2008/MA	JN808857	B	7.24%	7.24%	7.30%	7.30%	7.24%	7.24%	7.35%	7.24%	7.02%	6.97%	0.39%			
NiV/BD/HU/2008/RA	JN808863	B	7.24%	7.24%	7.30%	7.30%	7.24%	7.24%	7.35%	7.24%	7.02%	6.97%	0.39%	0.00%		
NiV/BD/HU/2010/FA1	JN808864	B	7.08%	7.08%	7.13%	7.13%	7.08%	7.08%	7.19%	7.08%	6.85%	6.80%	0.17%	0.55%	0.55%	
NiV/IN/HU/2007/FG	FJ513078	B	7.13%	7.13%	7.19%	7.19%	7.13%	7.13%	7.24%	7.13%	6.91%	6.85%	0.39%	0.44%	0.44%	0.55%

Technical Appendix Figure 5A. Estimates of evolutionary divergence between NiV G ORF nucleotide sequences. The number of base differences (top matrix) and percentage of difference (bottom matrix) per sequence from between sequences are shown. SE estimate(s) are shown above the diagonal. The analysis involved 15 nt sequences. Codon positions included were 1st+2nd+3rd+noncoding. There were a total of 1,809 positions in the final dataset.

Nipah sequence ID	Accession #	Genotype														
NiV/MY/HU/1999/CDC	AF212302	M														
NiV/MY/PI/1999/1413	AJ564622	M	0.00													
NiV/MY/PI/1999/2794	AJ564621	M	0.00	0.00												
NiV/MY/HU/1999/0128	AJ564623	M	0.00	0.00	0.00											
NiV/MY/HU/1999/UM1	AY029767	M	0.00	0.00	0.00	0.00										
NiV/MY/HU/1999/UM2	AY029768	M	0.00	0.00	0.00	0.00	0.00									
NiV/MY/BA/2000/TI	AF376747	M	0.00	0.00	0.00	0.00	0.00	0.00								
NiV/MY/PI/1999/0626	AJ627196	M	2.00	2.00	2.00	2.00	2.00	2.00	2.00							
NiV/MY/BA/2010/MY	FN869553	M	6.00	6.00	6.00	6.00	6.00	6.00	6.00	6.00						
NiV/KH/BA/2004/KHM	AY858111	M	9.00	9.00	9.00	9.00	9.00	9.00	9.00	11.00	9.00					
NiV/BD/HU/2004/RA1	AY988601	B	26.00	26.00	26.00	26.00	26.00	26.00	26.00	26.00	24.00	27.00				
NiV/BD/HU/2008/MA	JN808857	B	26.00	26.00	26.00	26.00	26.00	26.00	26.00	26.00	24.00	27.00	0.00			
NiV/BD/HU/2008/RA	JN808863	B	26.00	26.00	26.00	26.00	26.00	26.00	26.00	26.00	24.00	27.00	0.00	0.00		
NiV/BD/HU/2010/FA1	JN808864	B	27.00	27.00	27.00	27.00	27.00	27.00	27.00	27.00	25.00	28.00	1.00	1.00	1.00	
NiV/IN/HU/2007/FG	FJ513078	B	27.00	27.00	27.00	27.00	27.00	27.00	27.00	27.00	25.00	28.00	1.00	1.00	1.00	2.00

Nipah sequence ID	Accession #	Genotype														
NiV/MY/HU/1999/CDC	AF212302	M														
NiV/MY/PI/1999/1413	AJ564622	M	0.00%													
NiV/MY/PI/1999/2794	AJ564621	M	0.00%	0.00%												
NiV/MY/HU/1999/0128	AJ564623	M	0.00%	0.00%	0.00%											
NiV/MY/HU/1999/UM1	AY029767	M	0.00%	0.00%	0.00%	0.00%										
NiV/MY/HU/1999/UM2	AY029768	M	0.00%	0.00%	0.00%	0.00%	0.00%									
NiV/MY/BA/2000/TI	AF376747	M	0.00%	0.00%	0.00%	0.00%	0.00%	0.00%								
NiV/MY/PI/1999/0626	AJ627196	M	0.33%	0.33%	0.33%	0.33%	0.33%	0.33%	0.33%							
NiV/MY/BA/2010/MY	FN869553	M	1.00%	1.00%	1.00%	1.00%	1.00%	1.00%	1.00%	1.00%						
NiV/KH/BA/2004/KHM	AY858111	M	1.50%	1.50%	1.50%	1.50%	1.50%	1.50%	1.50%	1.83%	1.50%					
NiV/BD/HU/2004/RA1	AY988601	B	4.32%	4.32%	4.32%	4.32%	4.32%	4.32%	4.32%	4.32%	3.99%	4.49%				
NiV/BD/HU/2008/MA	JN808857	B	4.32%	4.32%	4.32%	4.32%	4.32%	4.32%	4.32%	4.32%	3.99%	4.49%	0.00%			
NiV/BD/HU/2008/RA	JN808863	B	4.32%	4.32%	4.32%	4.32%	4.32%	4.32%	4.32%	4.32%	3.99%	4.49%	0.00%	0.00%		
NiV/BD/HU/2010/FA1	JN808864	B	4.49%	4.49%	4.49%	4.49%	4.49%	4.49%	4.49%	4.49%	4.15%	4.65%	0.17%	0.17%	0.17%	
NiV/IN/HU/2007/FG	FJ513078	B	4.49%	4.49%	4.49%	4.49%	4.49%	4.49%	4.49%	4.49%	4.15%	4.65%	0.17%	0.17%	0.17%	0.33%

Technical Appendix Figure 5B. Estimates of evolutionary divergence between NiV G ORF amino acid sequences. The number of amino acid differences (top matrix) and percentage of difference (bottom matrix) per sequence from between sequences are shown. SE estimate(s) are shown above the diagonal. The analysis involved 15 aa sequences. Codon positions included were 1st+2nd+3rd+noncoding. There were a total of 602 positions in the final dataset.

Nipah sequence ID	Accession #	Genotype													
NiV/MY/HU/1999/CDC	AF212302	M													
NiV/MY/PI/1999/1413	AJ564622	M	1.0												
NiV/MY/PI/1999/2794	AJ564621	M	1.0	2.0											
NiV/MY/HU/1999/0128	AJ564623	M	2.0	3.0	1.0										
NiV/MY/HU/1999/UM1	AY029767	M	1.0	2.0	0.0	1.0									
NiV/MY/HU/1999/UM2	AY029768	M	1.0	2.0	2.0	3.0	2.0								
NiV/MY/PI/1999/0626	AJ627196	M	12.0	13.0	11.0	12.0	11.0	13.0							
NiV/MY/BA/2010/MY	FN869553	M	12.0	13.0	11.0	12.0	11.0	13.0	0.0						
NiV/BD/HU/2004/RA1	AY988601	B	441.0	440.0	441.0	442.0	441.0	442.0	446.0	446.0					
NiV/BD/HU/2008/MA	JN808857	B	435.0	434.0	435.0	436.0	435.0	436.0	440.0	440.0	53.0				
NiV/BD/HU/2008/RA	JN808863	B	435.0	434.0	435.0	436.0	435.0	436.0	440.0	440.0	53.0	0.0			
NiV/BD/HU/2010/FA1	JN808864	B	445.0	444.0	445.0	446.0	445.0	446.0	450.0	450.0	16.0	55.0	55.0		
NiV/IN/HU/2007/FG	FJ513078	B	432.0	431.0	432.0	433.0	432.0	433.0	437.0	437.0	47.0	19.0	19.0	49.0	

Nipah sequence ID	Accession #	Genotype													
NiV/MY/HU/1999/CDC	AF212302	M													
NiV/MY/PI/1999/1413	AJ564622	M	0.01%												
NiV/MY/PI/1999/2794	AJ564621	M	0.01%	0.03%											
NiV/MY/HU/1999/0128	AJ564623	M	0.03%	0.04%	0.01%										
NiV/MY/HU/1999/UM1	AY029767	M	0.01%	0.03%	0.00%	0.01%									
NiV/MY/HU/1999/UM2	AY029768	M	0.01%	0.03%	0.03%	0.04%	0.03%								
NiV/MY/PI/1999/0626	AJ627196	M	0.18%	0.19%	0.16%	0.18%	0.16%	0.19%							
NiV/MY/BA/2010/MY	FN869553	M	0.18%	0.19%	0.16%	0.18%	0.16%	0.19%	0.00%						
NiV/BD/HU/2004/RA1	AY988601	B	6.55%	6.53%	6.55%	6.56%	6.55%	6.56%	6.62%	6.62%					
NiV/BD/HU/2008/MA	JN808857	B	6.46%	6.44%	6.46%	6.47%	6.46%	6.47%	6.53%	6.53%	0.79%				
NiV/BD/HU/2008/RA	JN808863	B	6.46%	6.44%	6.46%	6.47%	6.46%	6.47%	6.53%	6.53%	0.79%	0.00%			
NiV/BD/HU/2010/FA1	JN808864	B	6.61%	6.59%	6.61%	6.62%	6.61%	6.62%	6.68%	6.68%	0.24%	0.82%	0.82%		
NiV/IN/HU/2007/FG	FJ513078	B	6.41%	6.40%	6.41%	6.43%	6.41%	6.43%	6.49%	6.49%	0.70%	0.28%	0.28%	0.73%	

Technical Appendix Figure 6A. Estimates of evolutionary divergence between NiV L ORF nucleotide sequences. The number of base differences (top matrix) and percentage of difference (bottom matrix) per sequence from between sequences are shown. SE estimate(s) are shown above the diagonal. The analysis involved 14 nt sequences. Codon positions included were 1st+2nd+3rd+noncoding. There were a total of 6,735 positions in the final dataset.

Nipah sequence ID	Accession #	Genotype												
NiV/MY/HU/1999/CDC	AF212302	M												
NiV/MY/PI/1999/1413	AJ564622	M	0.0											
NiV/MY/PI/1999/2794	AJ564621	M	1.0	1.0										
NiV/MY/HU/1999/0128	AJ564623	M	1.0	1.0	0.0									
NiV/MY/HU/1999/UM1	AY029767	M	1.0	1.0	0.0	0.0								
NiV/MY/HU/1999/UM2	AY029768	M	0.0	0.0	1.0	1.0	1.0							
NiV/MY/PI/1999/0626	AJ627196	M	4.0	4.0	3.0	3.0	3.0	4.0						
NiV/MY/BA/2010/MY	FN869553	M	4.0	4.0	3.0	3.0	3.0	4.0	0.0					
NiV/BD/HU/2004/RA1	AY988601	B	39.0	39.0	39.0	39.0	39.0	39.0	42.0	42.0				
NiV/BD/HU/2008/MA	JN808857	B	34.0	34.0	34.0	34.0	34.0	34.0	37.0	37.0	7.0			
NiV/BD/HU/2008/RA	JN808863	B	34.0	34.0	34.0	34.0	34.0	34.0	37.0	37.0	7.0	0.0		
NiV/BD/HU/2010/FA1	JN808864	B	40.0	40.0	40.0	40.0	40.0	40.0	43.0	43.0	3.0	10.0	10.0	
NiV/IN/HU/2007/FG	FJ513078	B	33.0	33.0	33.0	33.0	33.0	33.0	36.0	36.0	6.0	1.0	1.0	9.0

Nipah sequence ID	Accession #	Genotype												
NiV/MY/HU/1999/CDC	AF212302	M												
NiV/MY/PI/1999/1413	AJ564622	M	0.00%											
NiV/MY/PI/1999/2794	AJ564621	M	0.04%	0.04%										
NiV/MY/HU/1999/0128	AJ564623	M	0.04%	0.04%	0.00%									
NiV/MY/HU/1999/UM1	AY029767	M	0.04%	0.04%	0.00%	0.00%								
NiV/MY/HU/1999/UM2	AY029768	M	0.00%	0.00%	0.04%	0.04%	0.04%							
NiV/MY/PI/1999/0626	AJ627196	M	0.18%	0.18%	0.13%	0.13%	0.13%	0.18%						
NiV/MY/BA/2010/MY	FN869553	M	0.18%	0.18%	0.13%	0.13%	0.13%	0.18%	0.00%					
NiV/BD/HU/2004/RA1	AY988601	B	1.74%	1.74%	1.74%	1.74%	1.74%	1.74%	1.87%	1.87%				
NiV/BD/HU/2008/MA	JN808857	B	1.52%	1.52%	1.52%	1.52%	1.52%	1.52%	1.65%	1.65%	0.31%			
NiV/BD/HU/2008/RA	JN808863	B	1.52%	1.52%	1.52%	1.52%	1.52%	1.52%	1.65%	1.65%	0.31%	0.00%		
NiV/BD/HU/2010/FA1	JN808864	B	1.78%	1.78%	1.78%	1.78%	1.78%	1.78%	1.92%	1.92%	0.13%	0.45%	0.45%	
NiV/IN/HU/2007/FG	FJ513078	B	1.47%	1.47%	1.47%	1.47%	1.47%	1.47%	1.60%	1.60%	0.27%	0.04%	0.04%	0.40%

Technical Appendix Figure 6B. Estimates of evolutionary divergence between NiV L ORF amino acid sequences. The number of amino acid differences (top matrix) and percentage of difference (bottom matrix) per sequence from between sequences are shown. SE estimate(s) are shown above the diagonal. The analysis involved 14 aa sequences. Codon positions included were 1st+2nd+3rd+noncoding. There were a total of 2,244 positions in the final dataset.
